# The Immune Phenotype of Isolated Lymphoid Structures in Non-Tumorous Colon Mucosa Encrypts the Information on Pathobiology of Metastatic Colorectal Cancer

**DOI:** 10.3390/cancers12113117

**Published:** 2020-10-25

**Authors:** Felicitas Mungenast, Anastasia Meshcheryakova, Andrea Beer, Martina Salzmann, Dietmar Tamandl, Thomas Gruenberger, Peter Pietschmann, Oskar Koperek, Peter Birner, Ilan Kirsch, Harlan Robins, Martina Mittlboeck, Markus Jaritz, Michael Bergmann, Philip Zimmermann, Diana Mechtcheriakova

**Affiliations:** 1 Department of Pathophysiology and Allergy Research, Center for Pathophysiology, Infectiology and Immunology, Medical University of Vienna, 1090 Vienna, Austria; felicitas.mungenast@meduniwien.ac.at (F.M.); martina.salzmann@meduniwien.ac.at (M.S.); peter.pietschmann@meduniwien.ac.at (P.P.); 2 Department of Pathology, Medical University of Vienna, 1090 Vienna, Austria; andrea.beer@meduniwien.ac.at (A.B.); Koperek@labor-kaserer.at (O.K.); peter.birner@meduniwien.ac.at (P.B.); 3 Department of Biomedical Imaging and Image-Guided Therapy, Medical University of Vienna, 1090 Vienna, Austria; dietmar.tamandl@meduniwien.ac.at; 4 Department of Surgery, Kaiser Franz Josef Hospital, 1100 Vienna, Austria; thomas.gruenberger@gesundheitsverbund.at; 5 Adaptive Biotechnologies Corporation, Seattle, WA 98102, USA; lkirsch@adaptivebiotech.com (I.K.); hrobins@adaptivebiotech.com (H.R.); 6 Center for Medical Statistics, Informatics, and Intelligent Systems, Medical University of Vienna, 1090 Vienna, Austria; martina.mittlboeck@meduniwien.ac.at; 7 Research Institute of Molecular Pathology, Vienna Biocenter, 1030 Vienna, Austria; Markus.Jaritz@imp.ac.at; 8 Department of Surgery, Medical University of Vienna, 1090 Vienna, Austria; michael.bergmann@meduniwien.ac.at; 9 Nebion AG, 8048 Zürich, Switzerland; phz@nebion.com

**Keywords:** lymphoid structures, colonic mucosa, metastatic colorectal cancer, immuno-oncology, B lymphocytes, germinal center, tissue image cytometry, B-cell clonality, compendium-wide analysis, patient stratification strategy

## Abstract

**Simple Summary:**

Today, the presence of well-organized functional structures of immune cells at tumor sites, known as ectopic lymphoid structures, and their strong association with patient survival have been reported in more than ten different cancer types. We aimed to investigate whether there is a link between the patient-specific characteristics of pre-formed isolated lymphoid structures in non-tumorous colon tissue and the disease pathobiology for patients with metastatic colorectal cancer. The study employed a powerful approach of quantitative tissue image cytometry to compare lymphoid structures of different anatomical locations within the same patients. We showed that the properties of isolated lymphoid structures in non-tumorous colon tissue predefine the immune phenotype of ectopic lymphoid structures at primary and metastatic sites. We discovered that B-cell-enriched and highly proliferative lymphoid structures are prognostic towards an improved clinical outcome. The knowledge gained from this study expands our understanding of tumor-immune interactions and draws particular attention to the anti-tumor immune response guided by isolated lymphoid structures outside of tumor tissue.

**Abstract:**

The gut-associated lymphoid tissue represents an integral part of the immune system. Among the powerful players of the mucosa-associated lymphoid tissue are isolated lymphoid structures (ILSs), which as information centers, drive the local (and systemic) adaptive immune responses. Germinal center reactions, taking place within ILSs, involve the coordinated action of various immune cell types with a central role given to B cells. In the current study, we aimed at dissecting the impact of ILSs within non-tumorous colon tissue (NT) on the pathobiology of colorectal cancer (CRC) with metastasis in the liver (CRCLM). In particular, we focused on the immune phenotypes of ILSs and ectopic lymphoid structures (ELSs), built up at matching primary and metastatic tumor sites. We implemented an integrative analysis strategy on the basis of tissue image cytometry and clonality assessment to explore the immune phenotype of ILS/ELS at three tissue entities: NT, CRC, and CRCLM (69 specimens in total). Applying a panel of lineage markers used for immunostaining, we characterized and compared the anatomical features, the cellular composition, the activation, and proliferation status of ILSs and ELSs, and assessed the clinical relevance of staining-derived data sets. Our major discovery was that ILS characteristics at the NT site predefine the immune phenotype of ELSs at CRC and CRCLM. Thereby, B-cell-enriched (CD20) and highly proliferative (Ki67) ILSs and ELSs were found to be associated with improved clinical outcome in terms of survival and enabled patient stratification into risk groups. Moreover, the data revealed a linkage between B-cell clonality at the NT site and the metastatic characteristics of the tumor in the distant liver tissue. Consolidation of immunostaining-based findings with the results of compendium-wide transcriptomic analysis furthermore proposed CD27 as a novel marker of T follicular helper cells within lymphoid structures. Overall, the study nominates the ILS immune phenotype as a novel prognostic marker for patients with metastatic CRC.

## 1. Introduction

Cumulative data within the rapidly evolving field of immuno-oncology positions tumor-infiltrating B cells among powerful contributors to anti-tumor immunity [1,2,3,4]. The term “B cell” covers the heterogeneity of multifarious B-cell subsets and therewith reflects their unique roles and multifaceted modes of action. One considerable aspect that links B-cell biology to, the adaptive immunity, the inflammation process, and the tumor microenvironment is the unique ability of B cells to form ectopic lymphoid structures (ELSs; also known as tertiary lymphoid structures, TLSs) as multitasking information centers triggering local (and systemic) anti-tumor responses. This is an example of a complex biological system, where various cell populations of B-cell lineage, specialized T lymphocytes known as T follicular helper (TFH) cells, follicular dendritic cells (FDCs), and specialized high endothelial venules (HEVs) assemble and act together in a highly coordinated manner [5,6,7,8].

The microarchitecture of ELSs appears to have pronounced similarities with that of classical germinal centers (GC) established within secondary lymphoid organs. Active ELSs are positive for activation-induced cytidine deaminase (AID), the ultimate driver of class switch recombination (CSR) and somatic hypermutation (SHM) of immunoglobulin genes [9,10]. In cancer settings, the on-site products of ELS activity, such as tumor-instructed plasma cells as well as memory B cells, may have a direct impact on the pathobiology of disease. This not only means that high-affinity antibodies of various isotypes directed against local tumor (neo)-antigens are produced and ELS-related cytokines (or other bioactive mediators) are secreted to orchestrate immune and stromal cells as part of the tumor immune microenvironment; this furthermore strongly suggests the migration of memory B cells through blood circulation to distant tissues, including the hosting tissue for metastasis [11,12,13].

Today, the presence of ELSs, their strong prognostic value in terms of disease outcome and thereby patient stratification capability has been reported in more than ten different types of cancer [14,15,16,17], including primary colorectal cancer (CRC) and metastatic CRC in the liver (CRCLM), lung cancer, hepatocellular carcinoma, pancreatic cancer, and invasive breast cancer. A very recent discovery added heterogeneous soft-tissue sarcomas to the list of solid tumors where B-cell-enriched ELSs were shown to be the strongest prognostic factor associated with improved survival and a high response rate to PD1 blockage [18]. Various methodological approaches were applied to detect ELSs and link their characteristics to the clinical outcome [19,20,21,22,23,24,25,26,27,28,29]. Immunostaining procedures focused on markers such as CD20 (general B-cell marker), DC-LAMP (a marker for mature DCs), or PNAd (a marker of HEVs). Additively, recent developments in transcriptomics and immuno-bioinformatics identified particular gene signatures, which are able to predict the in-tissue presence of ELSs.

However, key information that needs to be considered for understanding ELSs in solid tumors is that there are tissue types where such lymphoid structures appear physiologically to fulfill an important balance between effective and protective immune response and self-tolerance. This holds true for organs with specialized immunity at epithelial barriers such as gastrointestinal, bronchopulmonary, and genitourinary mucosal barriers as well as skin [30,31,32]. The gut-associated lymphoid tissue (GALT) represents the paragon of mucosa-associated tissue, which is composed of special immune structures, including the cryptopatches, isolated lymphoid structures (ILSs), and Peyer’s patches/colonic patches. Those structures differ in their localization, size, and cellular composition [33,34,35,36,37].

Here, we address the question of the role of lymphoid structures in the pathobiology of metastatic CRC implementing a newly developed integrative strategy named DIICO/from Digital Immune Imaging to Clinical Outcome. Special focus was given to the investigation of ILSs in non-tumorous colon tissue located adjacent to the primary tumor (named herein as NT). Their role in CRC/CRCLM pathobiology has not yet been explored. We hypothesize that the immune phenotype of ILSs at the NT site predefines the anatomical features and the cellular architecture of ELSs at the CRC and/or CRCLM sites and thereby encrypt the information on disease outcome. This hypothesis is strongly supported by (i) the natural occurrence and the unique function of ILSs in the gut immunity and (ii) the discovery of functional ELSs with strong prognostic power at distant metastatic sites in the liver [38], the organ where B cells and B-cell-driven ELSs are physiological foreigners. The latter implies that the anti-tumor immune response at CRCLM site is pre-instructed at the primary CRC site and/or even within the NT tissue. The herein applied tissue image cytometry, which can be seen as the next-generation digital imaging or digital pathology approach, provided a detailed characterization of the anatomical features, cellular composition, proliferative and functional activity of ILSs and ELSs, built up at three locations (NT, primary CRC, and CRCLM) within a matched specimen collection. In the course of the quantitative analyses within DIICO, encrypted tissue information was transformed into numerical data for alignment with disease-relevant parameters. Additive information on critical ILS constituents was obtained by a comprehensive analysis of transcriptomic data and B-cell clonality assessment. In accordance with the hypothesis, our work adds ILSs as novel prognostic players orchestrating the pathobiology of CRCLM.

## 2. Results

### 2.1. Computerized Microscopy-Based Algorithm for Characterization of the Immune Phenotype of Lymphoid Structures 

**Figure 1:**
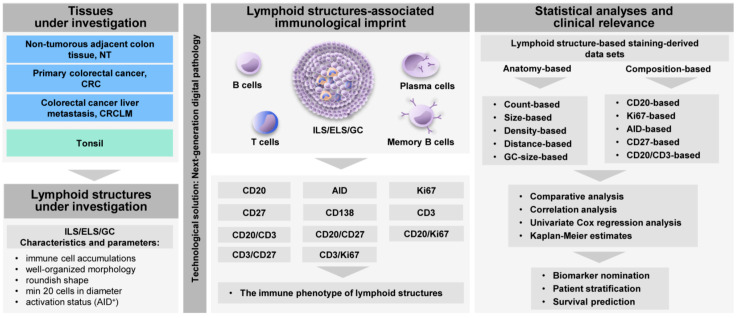
Next-generation digital pathology-based strategy for qualitative and quantitative analyses of the lymphoid structure-associated immunological imprint. Shown is the flow chart of the Digital Immune Imaging to Clinical Outcome (DIICO) strategy on the basis of data sets derived from the next-generation tissue image cytometry. This includes the tissues under investigation, the lymphoid structures under investigation and their characteristics, the set of profiling markers, the data-derived variables accounting for the spatial organization and anatomical features, the cellular composition as well as the functional activity of ILS/ELS. Additionally, given is an overview of the statistical evaluation with the main focus on the clinical relevance. ILS, isolated lymphoid structure; ELS, ectopic lymphoid structure; GC, germinal center; AID, activation-induced cytidine deaminase; NT, non-tumorous tissue; CRC, colorectal cancer; CRCLM, colorectal cancer liver metastasis.

**Figure 2:**
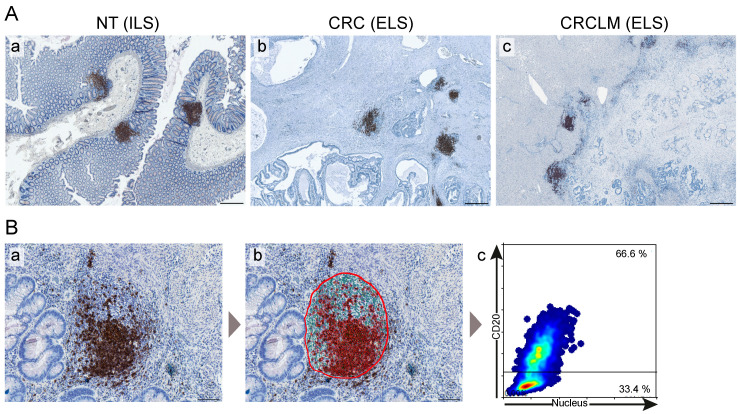
Lymphoid structures at three tissue entities of patients with metastatic colorectal cancer visualized by the digital image cytometry approach. (**A**) Representative images of ILS and ELS within tissue sections stained for the B-cell marker CD20 (**brown color**) and counterstained for nuclei with hematoxylin (**blue color**) are shown for three tissue entities (a, NT (ILS); b, CRC (ELS); c, CRCLM (ELS)). Scale bar: 500 µm. The whole-slide digital images were acquired using the automated microscopy-based TissueFAXS platform (TissueGnostics, Vienna, Austria). (**B**) The quantitative analysis of immune cell populations stained for a specific marker is based on a single cell recognition algorithm (nuclear segmentation) by HistoQuest software (TissueGnostics, Vienna, Austria). Shown are (a) the original image of ELS within CRC upon IHC staining for CD20 (brown color, CD20 staining; blue color, nuclear counterstaining with hematoxylin), (b) software-based recognition of CD20-positive cells (marked in **red**) and CD20-negative cells (marked in **green**) within the region of interest covering the entire ELS (indicated by a **red** circle) and (c) the corresponding scatter plot of the HistoQuest-based tissue image cytometry analysis showing the individual cells, represented by dots, detected within the ELS. The cut-off line separates the CD20-positive B cells (upper quadrant, 66.6%) from the CD20-negative cells (lower quadrant, 33.4%). Scale bar: 100 µm. NT, non-tumorous tissue; CRC, colorectal cancer; CRCLM, colorectal cancer liver metastasis; ILS, isolated lymphoid structure; ELS, ectopic lymphoid structure; IHC, immunohistochemical.

We established a next-generation digital pathology-based DIICO strategy (Figure 1) to assess the anatomical features, cellular composition, and activity of ILS/ELS assembled at three tissues sites of the same patient, the NT, CRC, and CRCLM sites (in total 69 samples). In accordance with the parameters and characteristics described in our previous study [38], we defined ILS/ELS as immune cell accumulations that have a well-organized morphology, a roundish shape, a minimum of 20 cells in diameter, and may have an AID-positive GC. The presence of ILS/ELS in NT, CRC, and CRCLM tissues is shown by using the B-cell marker CD20 (Figure 2A). To characterize the immune phenotype of ILS/ELS with a major focus given to B-cell subpopulations, we performed immunostaining of FFPE tissue sections for a set of markers (Figure 1). The panel included CD20 (a general marker for B cells), AID (a marker for functionally active GCs), CD27 (a marker for memory B cells, plasma cells, and for a subset of T cells), and CD138 (a marker for plasma cell). We additionally included CD3 (T-cell marker) and Ki67 (proliferation marker) into the marker panel to characterize the intra-follicular presence and distribution of the B-cell and T-cell zones and of proliferating immune cells. In addition, we visualized subpopulations by immunofluorescence (IF) double staining for the following combinations of markers: CD20/CD27 (memory B cells), CD3/CD27 (a subset of T cells), CD20/Ki67 (proliferating B cells), CD3/Ki67 (proliferating T cells), and CD20/CD3 (the B-cell/T-cell interplay within adaptive immune response). Tonsil tissue (*n* = 6) was used as a control tissue with established and functional GCs. Central to this study was the immune phenotyping of lymphoid structures and not the determination of the immune landscape throughout the entire mucosa/tumor/tumor-stoma tissue. Thus, software-based quantitative analysis of the magnitude of marker-positive subpopulations was performed within ILSs and ELSs, and were predefined as individual objects (Figure 2B). All ILS/ELS within a patient specimen were evaluated (as described in detail in Material and Methods). Applying the next-generation tissue image cytometry, encrypted tissue information was transformed into numerical data. As an outcome, staining-derived data sets, characterizing the patient-specific, lymphoid structure-associated immunological imprint, were obtained. The anatomy- and composition-based variables (*n* = 24) thereby included “ILS/ELS count”, “ILS/ELS size (mm^2^)”, “ILS/ELS density (cells/mm^2^)”, “GC size of ILS/ELS size (%)” (NT and CRC), “ILS to ILS distance (mm)” (NT), “CD20+ cells in ILS/ELS (%)”, “AID+ ILS/ELS count”, “Ki67+ cells in ILS/ELS (%)” and “CD27+ cells in ILS/ELS (%)” (Table S1). We used those data sets (i) to determine tissue type-specific differences and similarities of ILS/ELS characteristics of the matched samples, (ii) to find associations among the variables, both intra-tissue and inter-tissue, and (iii) to assess the clinical relevance in respect of survival prediction and patient stratification. The values represent the mean values calculated by the software across all lymphoid structures analyzed within a specimen.

### 2.2. The Immune Phenotype of ILS within NT

**Figure 3:**
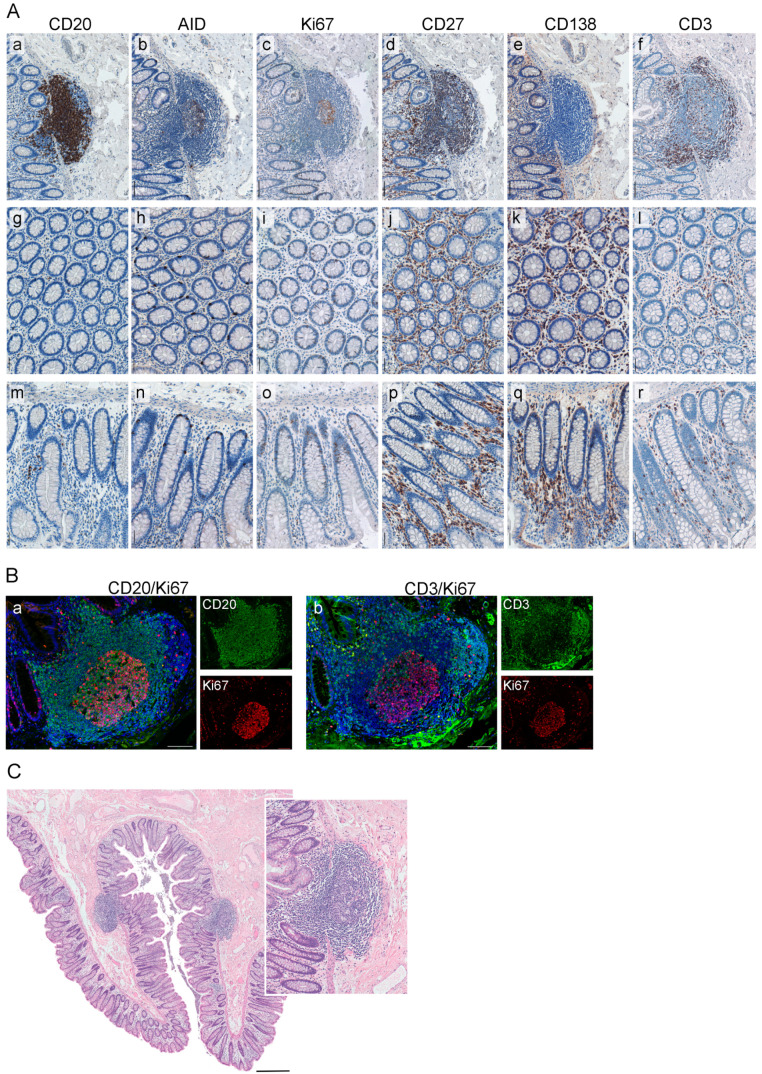
Characterization of the immune phenotype of ILS within NT. (**A**) Shown are the representative images of an ILSs within NT, and the corresponding colonic mucosa whereat the FFPE tissue sections were stained by IHC for the markers CD20 (a, g, m), AID (b, h, n), Ki67 (c, i, o), CD27 (d, j, p), CD138 (e, k, q), and CD3 (f, l, r); color code: **brown**, the marker; **blue**, nuclear counterstaining with hematoxylin. Scale bar: 100 µm. (**B**) The proliferative status of B-cell and T-cell populations within ILSs was assessed by IF staining. Color code: (a) **green**, CD20; **red**, Ki67; **blue**, nuclear counterstaining by DAPI; (b) **green**, CD3; **red**, Ki67; **blue**, nuclear counterstaining by DAPI. Shown are images for individual channels and the merged images. Scale bar: 100 µm. (**C**) The presence and localization of compact round-shaped ILSs within normal colon mucosa of NT is visualized by HE staining. Scale bar: 500 µm. Insert: enlarged view of ILS with prominent GC area. FFPE, formalin-fixed paraffin-embedded; IHC, immunohistochemical; AID, activation-induced cytidine deaminase; NT, non-tumorous tissue; ILS, isolated lymphoid structure; IF, immunofluorescent, HE, hematoxylin and eosin; GC, germinal center; DAPI, 4′,6-diamidino-2-phenylindole.

We applied a set of GC markers and the follow-up quantitative analyses and assessed the immune profiles of ILSs in non-tumorous colon tissue, NT, of CRCLM patients (Figure 3A and Table S2). Using CD20 as a marker, we demonstrated that the main constituents of ILS are B cells (median 70%). We further showed that the vast majority of the CD20-positive B-cell population within NT was present in the form of ILSs while only sparely distributed single CD20-positive cells were detected in the mucosal tissue (Figure 3A, a, g, m). Within the ILSs we detected the presence of an AID-positive core representing the functionally active GC of the lymphoid structure. AID-positive cells were thereby predominantly located in the dark zone of the GC. A similar distribution pattern within the ILS was observed for Ki67-positive/proliferating immune cells (Figure 3A, b, and c). Ki67-positive cells were not detected in the colonic mucosa outside the ILS (Figure 3A, i, o). Intriguingly, we found a strong presence of the CD27-positive cells (median 77%) within the entire ILS. Besides the ILSs, CD27-positive cell subsets were detected in the colonic mucosa (Figure 3A, j, p). Only single CD138-positive plasma cells were detected within the intra- or peri-follicular space; in contrast, CD138-positive cells can be easily detected in colonic mucosa between the crypts in the distance (> 1 mm) to ILSs (Figure 3A, e, k, q). CD3-positive T cells were detected within GC as well as within defined T-cell areas in the outer part of ILS. Contrary to CD20-positive B cells, CD3-positive T cells were additionally present in the colonic mucosa (Figure 3A, f, l, r). We next addressed the question regarding the proliferation status of B- and T-cell subpopulations. As revealed by the double staining approach applied to NT, the vast majority of Ki67-positive cells within GC of ILS is the CD20-positive B-cell population (Figure 3B, a). In contrast, only single Ki67-positive/CD3-positive cells were detected (Figure 3B, b). Additionally, we showed that ILSs can be identified and visualized by hematoxylin and eosin (HE) staining, followed by the computerized acquisition of whole-slide tissue sections (Figure 3C). Thereby, a NT sample may harbor up to 25 ILSs, with a median of 7 ILSs observed (Table S2).

Based on the profiled markers, we can conclude that the spatial organization and immune phenotype of ILSs in the colon showed visual similarities to the characteristics of the classical lymphoid structures in tonsils (Figure S1A,B). Comparative analysis based on the quantitative assessment revealed that parameters such as the size of the lymphoid structure, the cellular density, the GC size, the magnitude of CD20-positive cells, or Ki67-positive cells within the lymphoid structure were all significantly higher in tonsil tissue. On the contrary, the magnitude of CD27-positive cells was found to be significantly higher in the ILSs of NT (Figure S1C). This finding indicates that the functional activity of ILSs in colonic mucosa is directed towards the production of CD27-expressing memory cell subsets.

### 2.3. The Patient-Specific, Lymphoid Structure-Associated Immunological Imprint of NT, CRC, and CRCLM

**Figure 4:**
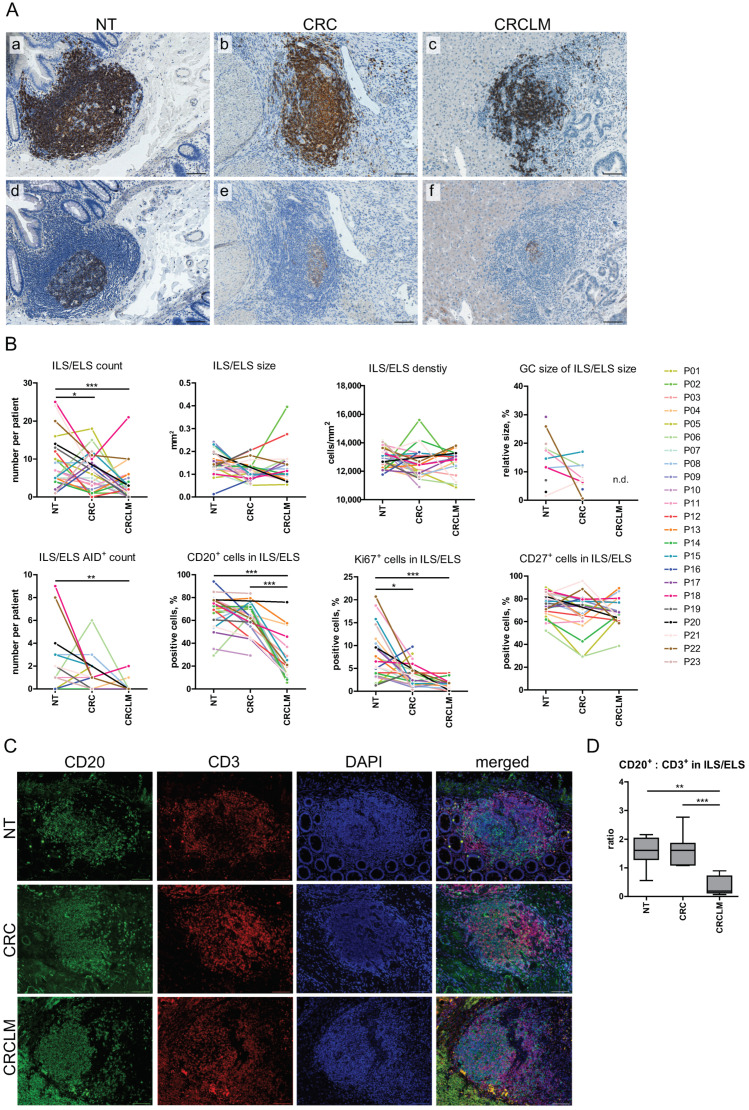
Comparative assessment of anatomical features, cellular compositions and activation status of ILS/ELS within NT, CRC and CRCLM. (**A**) Presence and functional activity of lymphoid structures were assessed at three tissue entities. Sequential tissue sections were stained for CD20 (a–c) and AID (d–f), (**brown** color, CD20 or AID staining; **blue** color, nuclear counterstaining with hematoxylin). Representative images of ILS/ELS with functionally active AID-positive GCs are shown within NT (a, d), CRC (b, e) and CRCLM (c, f). Scale bar: 100 µm. (**B**) The dot and line diagrams illustrate the comparisons of characteristics of ILS/ELS within NT, CRC, and CRCLM on the basis of three measurements per individual. All observations are plotted as individual dots, and the observations from different tissue entities of the same individual are connected by a line in a particular color. Comparisons were performed using Wilcoxons signed-rank test for pairwise group comparisons. Significant results are indicated by asterisks: * (*p* < 0.05), ** (*p* < 0.01), and *** (*p* < 0.001). Within ILS/ELS no or only single CD138-positive cells were detected; quantitative assessment of this marker was not performed. (**C**) Spatial distribution of CD20-positive B cells and CD3-positive T cells within ILS/ELS of NT, CRC, and CRCLM, assessed by double IF staining. Color code: **green**, CD20; **red**, CD3; **blue**, nuclear counterstaining by DAPI. Shown are images for individual and merged images. Scale bar: 100 µm. (**D**) Double staining images were used for quantitative analysis of the cellular composition of ILS/ELS in respect of the B-cell to T-cell ratio (NT, *n* = 7; CRC, *n* = 7; CRCLM, *n* = 9; in total, *n* = 23 specimens and 62 ILS/ELS). Boxplots illustrate the CD20/CD3 ratio at three tissue entities. Significant differences are indicated by asterisks ** (*p* < 0.01) and *** (*p* < 0.001) (Mann-Whitney-U test). NT, non-tumorous tissue; CRC, colorectal cancer; CRCLM, colorectal cancer liver metastasis; ILS, isolated lymphoid structure; ELS, ectopic lymphoid structure; AID, activation-induced cytidine deaminase; GC, germinal center; DAPI, 4′,6-diamidino-2-phenylindole; IF, immunofluorescent.

To our knowledge, we report for the first time the side by side comparison of the anatomical features, B-cell-attributed cellular composition, and activity of ILS/ELS at three tissue entities of the same patient (Table S1, Table S2, Figure 4, and Figure S2). Besides NT with the expected physiological appearance of ILSs, ELSs were detected at CRC and CRCLM sites of the analyzed cohort of patients (Figure 4A). This goes in line with previous studies [21,22,39,40,41], including one from our group [38], where we discovered lymphoid structures to be established precisely at the metastatic border. The number of organized lymphoid structures within a tissue, quantified as the ILS/ELS count, was found to be significantly higher in NT in comparison to CRCLM (*p* < 0.001) and in CRC in comparison to CRCLM (*p* = 0.038). Comparative analysis of variables attributed to the anatomical features of ILSs and ELSs such as ILS/ELS size, ILS/ELS density, and GC size of ILS/ELS did not reveal significant differences among the analyzed locations (Figure 4B). Functional activity of lymphoid structures, indicative for locally ongoing affinity maturation and class switch recombination processes, was first evaluated microscopically by the presence of AID-positive GC, as shown in Figure 4A and quantified as AID+ ILS/ELS count. NT showed the highest AID-positive ILS count (Figure 4B) with the prevalence of AID-positive ILS in 56% of patients. We found that in respect of CD20-positive B cells, ILSs in NT were similar to ELSs in CRC, both showing a strong prevalence of B cells (NT, median 70.19%, ranging from 29.30% to 94.04%; CRC, median 64.87%, ranging from 29.33% to 83.64%). CRCLM was characterized by a lower content of B cells within ELSs (median 19.86%, ranging from 5.69% to 75.94%).

We identified a significant difference in the proliferation status of ILS/ELS, measured by the magnitude of Ki67-positive cells, between NT and CRC as well as NT and CRCLM (*p* = 0.014 and *p* < 0.001, respectively), with the highest proliferation activity of immune cells located in the ILS within NT (median 5.40%, ranging from 1.28% to 20.71%). This suggests a reduced proliferative potential within the lymphoid structures detected at CRC and CRCLM sites.

As shown above (Figure 3), we discovered CD27 to be a novel marker of ILSs in NT. Quantitative assessment of the intra- and inter-tissue relationships revealed that the magnitude of CD27-positive cells within ILS/ELS gives a wide-ranged distribution: in NT, from 52.04% to 89.98% (median 77.34%); in CRC, from 28.57% to 95.68% (median 73.05%), and in CRCLM, from 38.75% to 89.38% (median 68.33%). However, no significant differences were found among the three tissue entities (Figure 4B). These data suggest that cells expressing CD27 are sustained as part of lymphoid structures in colonic mucosa, primary and metastatic CRC sites.

We further characterized the ILS/ELS cellular composition with respect to the relation between B cells and T cells by assessing their spatial distribution and content; the latter was measured by the CD20 to CD3 ratio. As can be seen in Figure 4C, lymphoid structures are characterized by the presence of B-cell and T-cell zones; additionally, CD3-positive T cells were diffusely distributed through GCs, likely representing the specialized TFH cells. In accordance with the above analysis, performed on the basis of the single staining-derived CD20-positive cell magnitude, we found a comparable CD20 to CD3 ratio among lymphoid structures of NT and CRC, characterized by a predominance of B cells (Figure 4D). The cellular composition differed for ELSs established at CRCLM site. Here, the quantitative analysis revealed a predominance of CD3-positive T cells.

### 2.4. The CD27-Positive Cell Populations within ILS Include TFH Cells 

**Figure 5:**
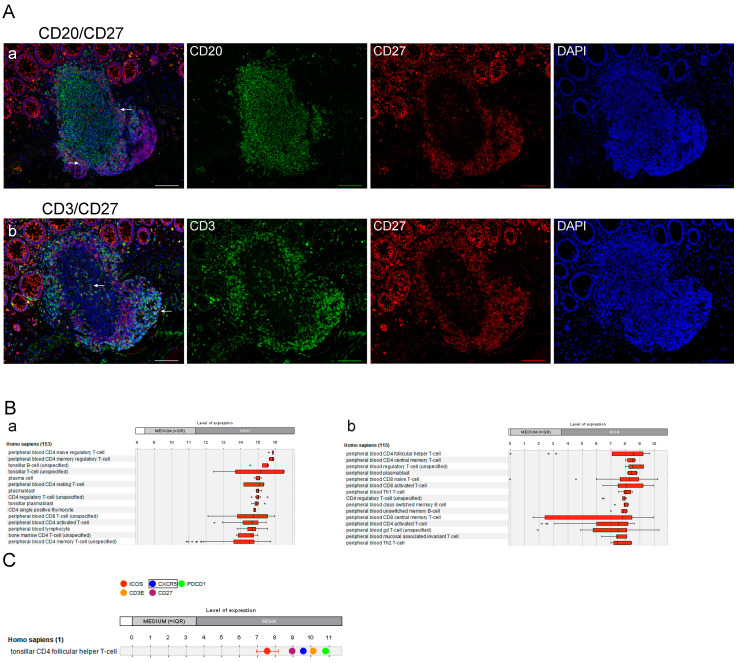
The CD27-associated immune phenotype of lymphoid structures and cell populations with the highest expression levels. (**A**) Localization of CD20/CD27 double-positive B cells (a) and CD3/CD27 double-positive T cells (b) within ILS in NT assessed by IF staining. Color code: **green**, CD20 or CD3; **red**, CD27; **blue**, nuclear counterstaining by DAPI. Shown are images for individual channels and the merged images. Examples of double-positive cells are indicated by white arrows. Scale bar: 100 µm. (**B**) GENEVESTIGATOR-based expression profile of the CD27 gene across publically available and curated microarray data sets from the Affymetrix Human Genome U133 Plus 2.0 Array platform (a) or mRNA-Seq Gene Level Homo sapiens (ref. Ensembl 75) platform (b). The filter “Anatomy_Cell Type_Healthy” included 7192 arrays (a) or 2471 arrays (b); conditions with *n* < 3 were excluded. The top 15 cell types showing the highest mRNA expression levels are listed; the expression profile is illustrated by the corresponding boxplot. Transcriptome data attributed to T follicular helper (TFH) cells was available only on the mRNA-Seq platform ((**B**), b, and (**C**)). (**C**) GENEVESTIGATOR-based expression profile of ICOS, CXCR5, PDCD1 (encoding PD1), CD3E, and CD27 in TFH cells. For this, the filter “Anatomy_Cell Type_Hematopoietic and Immune System Cell_T-cell lineage_Tonsillar CD4 Follicular Helper T-cell” was applied (study GSE58596). DAPI, 4′,6-diamidino-2-phenylindole; IF, immunofluorescent; TFH, T follicular helper; ICOS, inducible co-stimulator; CXCR5, C-X-C chemokine receptor type 5; PDCD1, programmed cell death protein 1.

Further information on the immune phenotype of ILSs in respect of CD27-positive cell populations was extracted from the analysis of CD20/CD27 and CD3/CD27 IF double staining combinations (Figure 5A). CD20/CD27 double-positive cells were distributed through the entire ILS (showing different levels of CD27 expression), including GC, and accumulated in the outer part of the lymphoid structure. ILSs were additionally characterized by the presence of CD3/CD27 double-positive cells which appear within GC, likely representing cells of the TFH lineage, and, besides, accumulate in the T-cell zone(s) proximate to ILSs. Such an immune phenotype is similar to one of the follicular structures within tonsil tissues (Figure S1A). In accordance with the observations made with respect to ILS, we show the presence of CD27-positive cells in ELSs at CRC and CRCLM sites (Figure S1B).

Next, we assessed the expression pattern of CD27 on the mRNA level using the compendium-wide analysis provided by the GENEVESTIGATOR platform (NEBION AG, Zurich, Switzerland). We used publicly available and manually curated data sets derived from a microarray and an RNA-Seq platform. Applying the filter for physiological conditions “Anatomy_Cell Type_Healthy” (*n* = 7192 for microarray data sets; *n* = 2471 for mRNA-Seq data sets), which covers a great variety of diverse cell populations of different origin, we found CD27 to be specifically expressed on immune cell populations at a high level (Figure 5B). The results from the two platforms were complementary as some of the cell populations/subpopulations are available on one of the platforms only. At the top positions were various T-cell subsets as well as tonsillar B cells, plasma cells and plasmablasts, and class-switched and unswitched memory B cells. These data once again emphasized that CD27 is a marker of immune cell populations, predominantly lymphocytes. Important among these findings is that the highest CD27 expression is attributed to TFH cells (Figure 5B, b). Combining this information with the localization and distribution pattern of CD3/CD27 double-positive cells in GCs (Figure 5A and Figure S3), we investigated the expression of CD27 simultaneously with established markers of TFH cells. As illustrated in Figure 5C, CD27 is expressed at high levels in tonsillar TFH cells along with the set of TFH-associated markers, including ICOS, CXCR5, PDCD1 (encoding PD1), and CD3E. This data confirms our hypothesis that CD27 can be nominated as a novel marker of TFH cells within lymphoid structures.

### 2.5. ILS Characteristics at NT Site Pre-Define the Immune Phenotype of ELS at CRC and CRCLM

**Figure 6:**
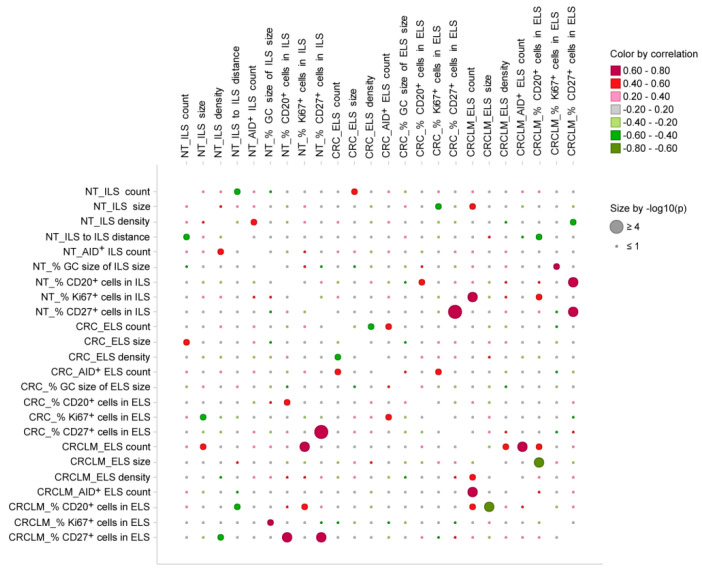
Interrelations among ILS/ELS-associated anatomy- and staining-derived variables. The bubble plot illustrates the Pearson correlation matrix-based associations (Table S3) among the ILS/ELS-associated variables (in total, *n* = 24 variables attributed to three tissue entities). The color indicates the strength and direction of the relationship between two variables based on the corresponding correlation coefficients; color code: **red**, positive correlation; **green**, negative correlation. Dot size is proportional to the p-value of correlation. Given the exploratory nature of the study, no correction for multiple testing was performed. NT, non-tumorous tissue; CRC, colorectal cancer; CRCLM, colorectal cancer liver metastasis; ILS, isolated lymphoid structure; ELS, ectopic lymphoid structure; AID, activation-induced cytidine deaminase; GC, germinal center.

From the results described above, we conclude that the magnitude, density, and cellular composition, as well as the activation and proliferation status of ILS/ELS varied among specimens and showed patient-specific characteristics. We next aimed to uncover associations among the anatomy- and staining-derived variables for each tissue individually and across the three tissue entities. Such analysis provides an additional layer of information on the local ILS/ELS-associated immune system and helps to embed ILS/ELS-attributed immune processes into a spatial context. It furthermore gives correlation-based predictions for fundamental relationships between the ILS/ELS at three locations. We found multiple strong intra- as well as inter-tissue correlations (correlation coefficient > 0.4; *p* < 0.05). Results are illustrated by a bubble plot (Figure 6) created on the basis of the correlation matrix (Table S3).

Among the most notable intra-tissue correlations, for NT we found positive associations between “NT_ILS density” and “NT_AID + ILS count”. This indicates that ILSs with a higher cellular density are able to establish a functionally active, AID-positive GC. Regarding CRC-based variables, positive associations were found between “CRC_ELS count” and “CRC_AID+ ELS count” and between “CRC_% Ki67+ cells in ELS” and “CRC_AID+ ELS count”. These likely suggest that as more ELSs were present at the primary tumor site as higher was the probability that those ELSs were functionally active/AID-positive. Furthermore, results indicate that ELS with higher proliferative status develop more often the GC-like AID-positive core, suggestive for the GC-reaction outcomes such as class-switched memory and/or plasma cells. For CRCLM, positive associations were found between “CRCLM_ELS count” and “CRCLM_ELS density”/“CRCLM_% CD20+ cells in ELS”/“CRCLM_AID+ ELS count”. These likely mean that a patient with a higher number of ELSs confined to the metastasis has a type of ELS which is characterized as structurally dense and B-cell-enriched and embeds a functionally active AID-positive core.

Analysis of the inter-tissue associations assesses interconnection between characteristics of ILSs in NT and ELSs in CRC and further distant ELSs in CRCLM. We found a positive association between “NT_% CD27+ cells in ILS” and “CRC_% CD27+ cells in ELS” and between “NT_% CD27+ cells in ILS” and “CRCLM_% CD27+ cells in ELS”. This likely indicates that the contexture of ILSs and ELSs with respect to the CD27-positive cell content was kept in all three tissue entities. For NT and CRC, positive correlations were found between the variables “NT_ILS count” and “CRC_ELS size” and between “NT_% CD20+ cells in ILS” and “CRC_% CD20+ cells in ELS”. Presumably, as more ILSs are present within NT as bigger the size of ELSs at the primary CRC site. This might be suggestive for a potential fusion process of neighboring ILSs, which take place in the course of cancer development or progression. Non-exclusively, this might suggest a more optimal situation for self-maintenance of ELSs at the CRC site. Furthermore, CRC-associated ELSs kept the magnitude of B cells predefined within ILSs at the NT site; those lymphoid structures were B-cell-enriched, as estimated by CD20 to CD3 ratio (Figure 4D). Importantly, for NT and the distant CRCLM site, multiple strong positive correlations were detected, such as those between “NT_ILS size” and “CRCLM_ELS count”, between “NT_% GC size of ILS size” and “CRCLM_% Ki67+ cells in ELS”, between “NT_% CD20+ cells in ILS” and “CRCLM_% CD27+ cells in ELS”, as well as between “NT_% Ki67+ cells in ILS” and “CRCLM_ELS count”/“CRCLM_% CD20+ cells in ELS”. The data propose a scenario where ILS characteristics, including the size, the GC size attributed to functional configuration, as well as the proliferative index, are pre-decisive for CD20-enriched (and CD27/memory phenotype-enriched) ELSs and for the number of ELSs at metastasis. Since both the magnitude of CD20-positive cells and the number of ELSs at the CRCLM border, according to our previous findings, have strong positive associations with the survival of patients with metastatic CRC [38], the data is strongly suggestive for a prognostic power of ILS characteristics and propose that the information encrypted in ILSs at NT site may predefine the disease outcome.

### 2.6. The Immune Phenotype of ILS within Non-Tumorous Colonic Mucosa Predicts Clinical Outcome of CRCLM Patients

**Figure 7:**
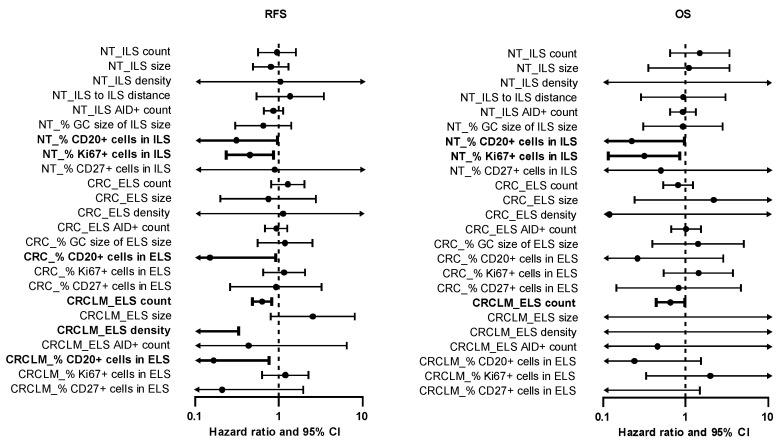
Prognostic power of staining-derived data sets estimated by Cox regression analyses. Forest plot shows the hazard ratio (circles) and 95% confidence intervals (horizontal bars). Statistically significant outcomes of univariate Cox regression analysis (Table S4) are highlighted in bold. Values exceeding the *x*-axis minima and maxima are ending with an arrow head. NT, non-tumorous tissue; CRC, colorectal cancer; CRCLM, colorectal cancer liver metastasis; ILS, isolated lymphoid structure; ELS, ectopic lymphoid structure; AID, activation-induced cytidine deaminase; GC, germinal center; CI, confidence interval; RFS, recurrence free survival; OS, overall survival.

To assess the clinical relevance of variables characterizing the ILS/ELS-associated immune phenotype, anatomy-/staining-derived data sets attributed to NT, CRC, and CRCLM sites were aligned with clinical outcome in terms of recurrence free survival (RFS) and overall survival (OS). Cox regression analysis (Figure 7 and Table S4) revealed that the variable “% CD20+ cells in ILS/ELS”, representing the B-cell fraction within ILS/ELS, showed a positive prognostic effect on RFS for all three tissue entities: NT (HR = 0.309, 95% CI: 0.099–0.966, *p* = 0.043), CRC (HR = 0.149, 95% CI: 0.024–0.923, *p* = 0.041), and CRCLM (HR = 0.166, 95% CI: 0.036–0.764, *p* = 0.021). This novel finding emphasizes the critical role of B cells as ILS/ELS constituents for the anti-tumor function of the entire lymphoid structures. Linking the herein discovered positive prognostic power attributed to ILSs in non-tumorous colonic mucosa to the above described correlation-based outcomes (Figure 6) positions ILS within NT as decisive biological structures orchestrating the immune responses at the primary and metastatic site. Another important finding was the positive association of the variable “NT_% Ki67+ cells in ILS” with RFS (HR = 0.450, 95% CI: 0.235–0.864, *p* = 0.016, Figure 7 and Table S4). This likely indicates that higher proliferative status of immune cells, as shown here predominantly of B cells (Figure 3B), within ILSs is linked to favorable disease outcome. Furthermore, interpreting this finding in the context of correlation-based interrelation with ELSs in CRCLM (Figure 6), we propose the proliferation status of ILS-associated cells to be decisive for the anti-tumor potential of lymphoid structures at the metastatic site. In accordance with our previous findings [38], we found positive prognostic effects for the CRCLM-attributed variables “CRCLM_ELS count” (HR = 0.629, 95% CI: 0.482–0.820, *p* < 0.001) and “CRCLM_ELS density” (HR < 0.001, 95% CI: < 0.001–0.330, *p* = 0.026). For OS, a significant association was found for the NT-related variables “NT_% CD20+ cells in ILS” (HR = 0.222, 95% CI: 0.051–0.957, *p* = 0.044) and “NT_% Ki67+ cells in ILS” (HR = 0.314, 95% CI: 0.115–0.854, *p* = 0.023) and for the CRCLM-related variable “CRCLM_ELS count” (HR = 0.652, 95% CI: 0.437–0.974, *p* = 0.037) (Figure 7 and Table S4).

**Figure 8:**
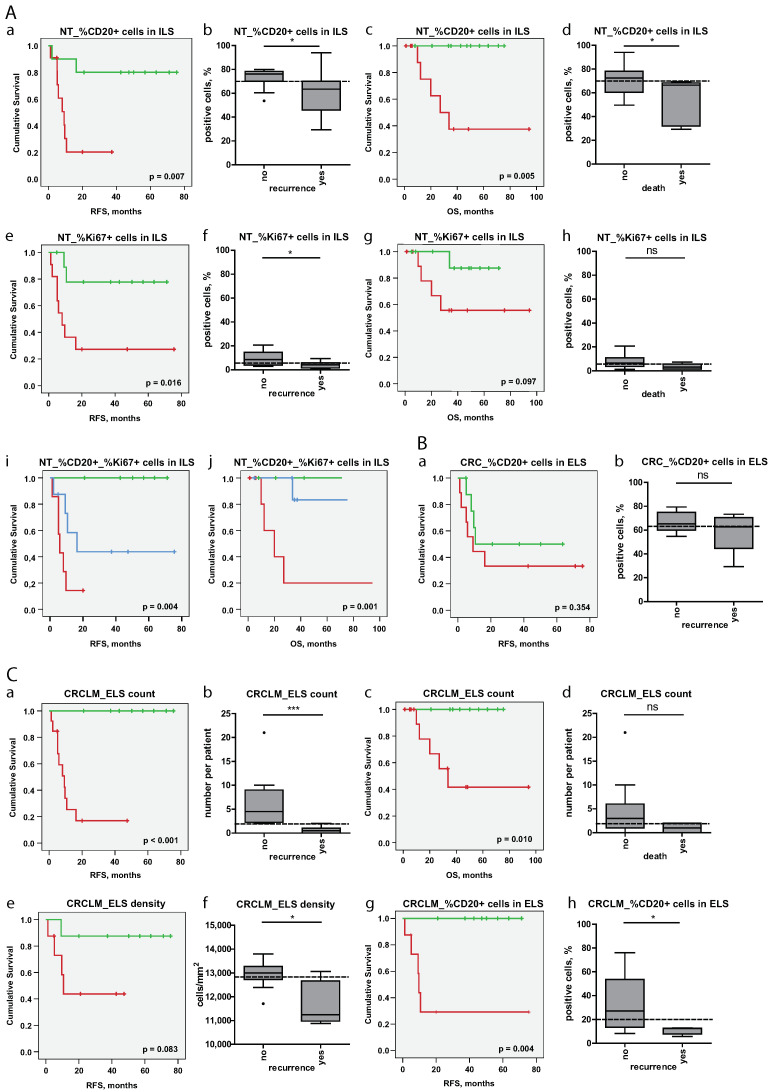
Patient stratification strategies on the basis of staining-derived data sets. Shown are Kaplan-Meier estimates for patient stratification in terms of survival based on staining-derived variables attributed to NT ((**A**), a, c, e, g, i, and j), CRC ((**B**), a), and CRCLM ((**C**), a, c, e, and g) tissue entities. RFS and OS were shown by Kaplan-Meier graphs, stratified by the median of the corresponding variable (Table S2) into low- and high-risk groups (higher than median indicates lowrisk); *p*-value of the log-rank test is indicated. Additionally, the combined variable “NT_% CD20+_% Ki67+ cells in ILS” was used for patient stratification into low-risk group (classified as low/low risk based on the variables “NT_% CD20+ cells in ILS” and “NT_% Ki67+ cells in ILS”), intermediate-risk group (classified as low risk based on the first variable and as high risk based on the second variable and vice versa), and high-risk group (classified as high/high risk based on the variables “NT_% CD20+ cells in ILS” and “NT_% Ki67+ cells in ILS”). Furthermore, the boxplots ((**A**), b, d, f, and h for NT; (**B**), b for CRC; C, b, d, f, and h for CRCLM) illustrate the sub-division of patients without event (disease recurrence in case of RFS and death in case of OS) and patients with event, named as “no” and “yes”, respectively. This was used as an additional exploratory representation of data; the group membership may change over time. Herein, the median value, which was used for patient stratification on the corresponding Kaplan-Meier plots, is indicated by the dashed line. Mann-Whitney-U test was used for group comparison. Significant results are indicated by asterisks: * (*p* < 0.05) and *** (*p* < 0.001); ns, not significant. RFS, recurrence free survival; OS, overall survival; NT, non-tumorous tissue; CRC, colorectal cancer; CRCLM, colorectal cancer liver metastasis; ILS, isolated lymphoid structure; ELS, ectopic lymphoid structure.

For all variables showing significant association with survival in univariate Cox regression analyses, Kaplan-Meier estimates were created to stratify patients into risk groups (Figure 8). Statistically significant stratifications into low-risk and high-risk groups were found for the variables attributed to NT (Figure 8A) such as “NT_% CD20+ cells in ILS” (log-rank test: *p* = 0.007, RFS and p = 0.005, OS) and “NT_% Ki67+ cells in ILS” (log-rank test: *p* = 0.016, RFS). For CRCLM-associated variables (Figure 8C), statistical significance was reached for “CRCLM_ELS count” (log-rank test: *p* < 0.001, RFS and *p* = 0.010, OS) and “CRCLM_% CD20+ cells in ELS” (log-rank test: *p* = 0.004, RFS). In addition to the above described single variables with significance, the combination of the variables “NT_% CD20+ cells in ILS” and “NT_% Ki67+ cells in ILS” named as “NT_% CD20+_% Ki67+ cells in ILS” (Figure 8A) was used for the creation of Kaplan Meier estimates. This combination showed significant patient stratification into low-, intermediate-, and high-risk groups in respect of both RFS and OS (log-rank test: *p* = 0.004, RFS and *p* = 0.001, OS).

In this analysis, we identified the relationships between the immune phenotype of ILSs and ELSs and the disease outcome in terms of survival. Notably, CD20-enriched (B-cell-enriched) ILS/ELS at all three locations showed positive prognostic power in respect to RFS. An additional characteristic that evolved to be prognostically important was the proliferation status of lymphoid structures. A major discovery to highlight herein is the prognostic effect of the immune phenotype of ILSs, located within non-tumorous colonic mucosa, on the survival of CRCLM patients.

### 2.7. Linkage between B-cell Clonality at NT Site and the Metastatic Characteristics of the Tumor in the Distant Hosting Liver Tissue

We performed an assessment of the B-cell clonality by analyzing Ig gene rearrangements within Ig heavy chain (IGH), Ig kappa and lambda (IGKL) in FFPE tissues attributed to NT, CRC, and CRCLM entities. The major limiting factor of clonality assessment from FFPE tissues was found to be attributed to an insufficient number of cells under investigation, herein infiltrating B cells, present within the analyzed tissue. As evaluated, more than 3400 CD20-positive B cells within a tissue are required to enable clonality assessment. Due to this methodological cut-off in sensitivity, the clonality of B cells could not be successfully evaluated in CRCLM specimens.

**Figure 9:**
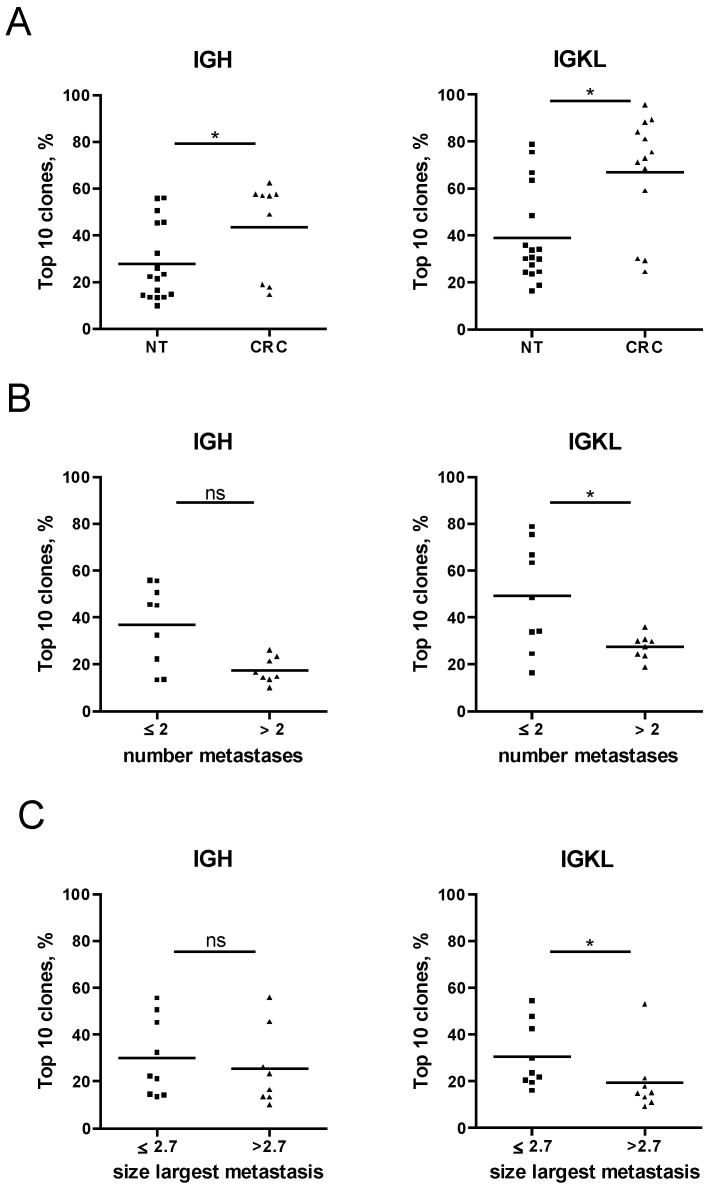
Assessment of B-cell clonality in FFPE tissues and the association with the metastatic behavior of the tumor. B-cell clonality was assessed by analyzing DNA, extracted from FFPE tissues for IGH and IGKL rearrangements. On the basis of the variable “Top 10 clones, %” the B-cell repertoire was compared between NT specimens and CRC specimens (**A**), between specimen grouped based on the number of metastases (**B**), and between specimens grouped based on the size of the largest metastasis (**C**). Only samples with at least 30 productive templates were included in the analysis. Group differences were assessed by Mann-Whitney-U test. Significant results are indicated by asterisks: * (*p* < 0.05); ns, not significant. NT, non-tumorous tissue; CRC, colorectal cancer; IGH, Ig heavy chain; IGKL, Ig kappa, and lambda, FFPE, formalin-fixed paraffin-embedded; ns, not significant.

The outcome of the analysis was presented by the variable “Top 10 clones, %”, which describes the proportion of the total clonal repertoire attributed to the top 10 clones; a higher proportion (close to 100%) is characteristic for a monoclonal/oligoclonal sample. The proportion of the IGH and IGKL Top 10 clones was found to be significantly higher in CRC specimens than in NT specimens (Figure 9). This likely indicates that a more polyclonal B-cell repertoire within NT was shifted toward a more oligoclonal type at the CRC site. We further aligned the clonality data of NT with clinicopathological parameters. We found an association of clonality repertoire with parameters such as “number of liver metastases” and “size of the largest liver metastasis”. Thereby the patient group with a lower number of metastases (≤2) and/or the smaller size of the largest metastasis (≤2.7 cm) was found to be characterized by a less diverse, oligoclonal B-cell repertoire, whereas the group with a higher number of metastases (>2) and/or the larger size of the largest metastasis (>2.7 cm) showed polyclonal characteristics (Figure 9). These important findings suggest a linkage between B-cell responses in terms of clonality at the non-tumorous colon mucosa and the metastatic behavior of the tumor.

Next we performed correlation analysis to find associations of the variable “Top 10 clones, %” from NT and the ILS/ELS-attributed anatomy-/staining-derived data sets. Strong significant positive association was found between “Top 10 clones_IGH, %” and “CRC_% GC size of ELS size” (correlation coefficient = 0.756; *p* = 0.049)/“CRC_% CD20+ cells in ELS” (correlation coefficient = 0.514; *p* = 0.041) and between “Top 10 clones_IGKL, %” and “CRC_% GC size of ELS size” (correlation coefficient = 0.786; *p* = 0.036)/“CRC_% CD20+ cells in ELS” (correlation coefficient = 0.515; *p* = 0.041)/“CRC_AID+ ELS count” (correlation coefficient = 0.500; *p* = 0.041). This strongly underlines that characteristics of the B-cell-attributed immune response at NT site influence the immune phenotype of ELSs at the tumor site. Thereby the oligoclonal nature of the B-cell repertoire within non-tumorous mucosa shows an association with B-cell enrichment of ELSs, and this is in turn a variable that was found to be a positive prognostic factor for RFS (Figure 7 and Table S4).

## 3. Discussion

Encouraged by our original discovery of the strong prognostic relevance of ELSs, which are established precisely at the metastatic border of CRC-derived metastasis in the liver [38], we continued to elucidate the immune phenotype of lymphoid structures and its interrelation with the pathobiology of metastatic CRC. Based on the outcome of our first study [38], we initially hypothesized that the locally formed structures around metastasis might be pre-instructed at the primary CRC tumor site. However, the results of the current pilot study are strongly suggestive for the interconnection between the immune phenotype of ILSs within non-tumorous colon tissue and of those lymphoid structures built up at primary and metastatic sites. In terms of clinical relevance, we identified that ILS characteristics might encrypt the information on disease outcome. The ILS-based assessment is strongly complementary to the knowledge derived from the analysis evaluating the Immunoscore [42,43].

Our study positions B cells as critical constituents of ILSs and ELSs. A major discovery to highlight is that the B-cell-enriched immune phenotype of ILSs within NT is associated with improved prognosis of CRCLM patients and that the variable characterizing the magnitude of CD20-positive cells within ILS can be used as a marker to stratify patients into risk groups. We would like to emphasize that CD20-positive B cells are exclusively localized within ILSs in NT as compared to the heterogeneous T-cell distribution pattern, which is not tied to ILS. Consequently, CD20 as a marker is highly specific for those lymphoid structures within colonic mucosa. We propose CD20 as a unique molecular identifier to define the presence of ILSs by immunostaining; this also holds true for gene expression profiling of non-tumorous colonic tissue for MS4A1 encoding CD20. Additionally important for the CRCLM prognosis is the proliferative status of B cells within the AID-positive GC of ILS. The Ki67-positive GC is indicative of the presence of active and mature lymphoid structures. The findings are consistent with a recent discovery emphasizing the favorable role of the Ki67^high^ B-cell population within TLS of melanoma patients [44]. Moreover, besides ILSs, we herein showed that the B-cell-enriched phenotype of ELSs at primary CRC and CRCLM sites also associates with a better clinical outcome in terms of survival.

While ILSs being a part of specialized natural immunity at the epithelial barrier in the colon are formed early after birth and are strongly influenced by gut-associated microbiota [33], the disease-driven formation of ELSs at metastasis indicates that the driver antigen(s) is present locally and recognized by the immune system. The herein uncovered similarity between the immune phenotypes of ILSs and ELSs in terms of their cellular composition, activation, and proliferation status may intriguingly indicate that the inducing antigen(s) is already present and recognized within the colonic mucosa of non-tumorous tissue. The findings further suggest that the capability to maintain and/or establish the corresponding adaptive immune response at the site of the tumor and/or the metastasis in the form of ELSs is patient-specific and acts as the critical determinant of disease progression and outcome. The described discoveries give us an opportunity to look at 3E theory, presented by Robert Schreiber now more than a decade ago, describing the role of the immune system in tumor “elimination”, “equilibrium”, and “escape” [45], in a new light. One would assume that the development of metastatic lesions is consistent with “escape”, but this study seems to somewhat complicate that formulation.

Our study gives special attention to the CD27 molecule. Initially, we aimed to use CD27 as a marker of memory B cells (moderate expression) and plasma cells (high expression) and subsets of memory T cells [46,47,48] and by this characterize the outcome of the local activity of the GC reaction. We found that the vast majority of cells of different lineages within ILSs are CD27-positive. This includes the CD20/CD27-positive B cells as well as CD3/CD27-positive T cells. This observation is suggestive of a key role of this regulatory molecule in the biology, maintenance, and functionality of ILSs in the colon. This conclusion is further supported by the finding that the magnitude of CD27-positive cells is significantly higher in ILSs in comparison to lymphoid structures located in tonsils. The immunostaining pattern of CD3/CD27-positive T cells within the GC area was suggestive of their TFH cell phenotype. To gain a deeper molecular understanding, we performed a compendium-wide analysis of CD27 in relation to the set of known markers for TFH cells across mRNA-seq data sets. Cumulative knowledge gained from immunostaining and transcriptomic analyses propose CD27 as a novel marker for TFH cells. In sum, our CD27-related findings bring attention to this molecule as a novel target allowing modulation of ILS-driven anti-tumor immune responses. A potential question for future analysis is whether agonistic anti-CD27 antibodies [49,50] may potentiate the intrinsic power of ILSs in the colon.

Another important finding interrelates the metastatic behavior of the tumor, represented by the number and size of metastases, with the B-cell clonality repertoire at the NT site. This important additional evidence highlights the characteristics of the GALT within the colonic mucosa as a decisive biological component orchestrating anti-tumor immune responses in the course of disease. With respect to clonality, a recent study of Zhang et al. [51] demonstrated that the clonality repertoire is spatially homogeneous across various distant colonic segments. Consequently, in the light of this data, we may conclude that the characterization of the herein described lymphoid structure-associated immune phenotype can be performed without accounting for the anatomical localization of the ILS.

In terms of translational perspectives, characterization of ILS-associated immune phenotype in non-tumorous colon tissue adjacent to the primary tumor offers a highly advantageous standardizing benefit in comparison to the investigation of the immunological imprint at the primary CRC site, given the potential heterogeneity of the immune contexture and immune landscape of the individual tumor parts. By analyzing the NT specimen, which is routinely available upon surgical operation [52], the influence of spatial heterogeneity across primary CRC tissue can be avoided. At the metastatic site in the liver, the standardization in assessment is guaranteed by the defined region for quantitative analysis, represented by the tumor-liver border [38]. The challenging feature of lymphoid structures for multiplex immunostaining-based quantitative analysis is the dense and compact cellular arrangement of distinct immune cell populations, which are physically interacting within ILS. To ensure proper quantification of more than one stained marker simultaneously, the question of touching objects/cells needs to be resolved first. This is among the prioritized challenges addressed in the field of tissue image cytometry, and there are promising indications that the algorithm might be released soon.

## 4. Materials and Methods 

### 4.1. Profile of Study Patients

This was an exploratory retrospective study on the pathobiology of CRCLM. Included were tissue samples collected from a cohort of 23 patients diagnosed with CRCLM, who underwent surgery at the Department of Surgery, Medical University of Vienna. The inclusion criterion and the sample size were determined by the availability of matched samples, in the form of paraffin-embedded tissue blocks obtained from three different tissue entities from each patient (in total, *n* = 69), and of clinicopathological data. Included tissues are from NT, CRC, and CRCLM sites. The NT tissue was obtained at the resection margin and represents the colon tissue, which is located at a distance of more than 5 cm apart from the primary tumor.

Within the patient cohort, 14 patients (61%) were male, and nine patients (39%) were female. The median age at the time of diagnosis of CRCLM was 65 years (ranging from 46 to 79). The median follow-up time was 47.34 months (95% CI: 28.70–65.98 months). A more detailed characterization of the patient population is provided in Table S5 and [38]. The patient cohort included 15 patients that underwent resection without preoperative chemotherapy. The remaining patients received fluoropyrimidine-based cytotoxic chemotherapy in combination with oxaliplatin and bevacizumab treatment prior to liver resection. Overall survival (OS) was defined as the time between diagnosis of CRCLM and cancer-related death (number of events, *n* = 5) and recurrence free survival (RFS) as the time between diagnosis of CRCLM and disease progression in the form of recurrence of metastasis or any type of tumor (number of events, *n* = 10).

### 4.2. Ethical Approval and Consent to Participate

The study was approved by the Ethics Committee of the Medical University of Vienna (EK-Nr. 1101/2013, including Amendment from 8 February 2016 and 15 December 2016). The informed consent was waived by the institutional review board due to the retrospective nature of the study.

### 4.3. Immunohistochemical and Immunofluorescence Staining of Formalin-Fixed Paraffin-Embedded Tissue Sections

Formalin-fixed paraffin-embedded (FFPE) tissue sections, 4 µm thick, of NT, CRC, and CRCLM underwent routine staining with hematoxylin and eosin (HE) to visualize the tissue morphology. Consequent sections were stained for the markers CD20, AID, Ki67, CD27, CD138, and CD3 (Figure 1) according to the protocols and validation strategies described by us previously [38,53,54]; the latter included tonsils as control tissue type composed of diverse populations of immune cells and harboring established GCs and the use of the GENEVESTIGATOR platform (NEBION AG, Zurich, Switzerland). To detect CD20, the general B-cell marker, we used either the clone L26 (mouse monoclonal antibody, Thermo Fisher Scientific, Runcorn, UK) or E17-P (rabbit clonal antibody, DB Biotech, Kosice, Slovak Republic). AID, the marker of activated B cells and the functionally active GCs, was detected with the clone ZA001 (mouse monoclonal antibody, Invitrogen by Thermo Fisher Scientific, Paisley, UK). For detection of Ki67, the marker of proliferating cells of either origin, Ab15580 antibody (rabbit polyclonal antibody, Abcam, Cambridge, UK), or clone UMAB107 (mouse monoclonal antibody, OriGene Technologies, Herford, Germany) were used. For identification of the CD27 molecule, the clone 137B4 (mouse monoclonal antibody, Thermo Fisher Scientific, Runcorn, UK) was used. For CD3, the marker of T cells, Ab5690 (rabbit polyclonal antibody, Abcam, Cambridge, UK) was used.

Within the IHC-based protocol for CD20, AID, Ki67, CD138, and CD3 DAKO EnVision+ Peroxidase system (DAKO, Glostrup, Denmark), and for CD27, the SignalStain Boost IHC Detection Reagent system (CellSignaling Technology Europe, Leiden, The Netherlands) were applied. The tissue sections were counterstained with hematoxylin (DAKO, Glostrup, Denmark). The IF double staining was performed using secondary goat anti-mouse/rabbit antibodies labeled with Alexa Fluor 488 and Alexa Fluor 568 dyes (Invitrogen, Paisley, UK). To enhance the IF signal for CD27, the biotinylated goat anti-mouse antibody (Life Technologies by Thermo Fisher Scientific, Runcorn, UK) in combination with streptavidin AF568 (Thermo Fisher Scientific, Runcorn, UK) was used. For visualization of the cell nuclei in IF, DAPI (Roche, Mannheim, Germany) was applied. The double staining was performed on tissue sections of a sub-group of patients, characterized by the presence of functionally active, AID-positive ILS/ELS at either location.

### 4.4. Quantitative Tissue Image Cytometry for Evaluation of Lymphoid Structure-Associated Immunological Imprint

We used the next-generation tissue image cytometry, the TissueFAXS platform (TissueGnostics, Vienna, Austria), for automatic acquisition (TissueFAXS imaging software versions 4.2.6245.1019 and 6.0.6245.123) and follow-up analyses of the IHC- and IF-stained whole-slide tissue sections. The analysis algorithm is based on a single cell recognition strategy and the quantification of the immune marker-positive cells using HistoQuest (for IHC, versions 3.5.3.0188, 4.0.4.0159 and 6.0.1.125) and TissueQuest (for IF, version 4.0.1.0128) software packages (TissueGnostics, Vienna, Austria). For each tissue type and marker under investigation, a specific profile was developed and applied, as described in detail previously [38]. Each lymphoid structure (ILS or ELS) in a specimen was considered by the software as an individual object for the quantitative analysis of marker-positive cells. All lymphoid structures identified within NT or primary CRC tissue were included in the analysis. For CRCLM, ELSs present at the tumor-liver border were analyzed as defined by us previously in [38]. For each specimen and a given tissue entity, a mean value was calculated by the software across the lymphoid structures included in the analysis. Thus, for each patient, three values per marker attributed to NT, CRC, and CRCLM were obtained. As an outcome, anatomy-/staining-derived data sets, characterizing the patient-specific, lymphoid structure-associated immunological imprint, were extracted. Those data sets were used as variables for statistical analysis and evaluation of clinical relevance.

### 4.5. Assessment of the Immunoglobulin Repertoire of Tumor-Infiltrating/Resident B cells

We performed a clonality assessment by analyzing Ig gene rearrangements within Ig heavy chain (IGH), Ig kappa, and lambda (IGKL). The source of DNA for analysis was FFPE tissue. The same FFPE tissue blocks, which were used for preparing the tissue sections for staining of markers described above, were used for clonality assessment. Thus, each specimen was fully characterized in terms of the total number of ILS/ELS, the number of AID-positive ILS/ELS, and the magnitude of CD20-positive B cells, the proliferative index of cells within ILS/ELS, as well as the clinicopathological parameters. This gives an indisputable advantage for sophisticated data analysis and interpretation. FFPE sections (25 µm) from 18 patients of the three entities (NT, CRC, and CRCLM) were included in the analysis (in total 56 samples, including two tonsil tissues as positive control). The B-cell clonality assessment was performed by Adaptive Biotechnologies (Seattle, WA, USA) with the ImmunoSEQ Assay for IGH and IGKL.

### 4.6. Compendium-Wide Analysis of the Expression Pattern of CD27 and the Markers of TFH Cells Across Transcriptomic Data Sets

We complemented the staining-based analysis of the CD27 protein by detailed characterization of the expression profile on the mRNA level. The GENEVESTIGATOR platform (NEBION AG, Zurich, Switzerland) [55]), which integrates publicly available and manually curated microarray and RNA sequencing data sets, was used to extract the expression pattern of CD27 across various cell types. The integrative analysis algorithm was described by us in detail previously [53,56,57]. A selection of healthy human samples was made for the Affymetrix Human Genome U133 Plus 2.0 Array compendium and likewise for the Illumina mRNA-Seq (ref. Ensembl 75) compendium. The analysis was performed on each platform separately. The top 15 outcomes (ranked by the gene expression levels) were exported. Furthermore, the mRNA expression levels of CD27, ICOS, CXCR5, PDCD1, and CD3E were assessed in TFH cells using the above mRNA-Seq compendium.

### 4.7. Statistical Analysis and Data Visualization 

ILS/ELS-associated anatomy and staining-derived variables were represented by a value that was calculated by the tissue image cytometry software as the mean across all lymphoid structures in a given tissue. Continuous variables were described with median, minimum, and maximum. Group differences among non-paired samples were assessed by nonparametric Kruskal-Wallis test for overall significance tests and Mann-Whitney-U test for pairwise group comparisons. Only in case of overall significance, pairwise comparisons by Mann-Whitney-U test were performed. Group differences among paired samples were assessed using the Wilcoxons signed-rank test for pairwise comparisons. Given the exploratory nature of the study, no corrections for multiple testing were performed. To achieve approximate normal distribution, staining-derived values were log2 transformed. Univariate Cox regression analysis was performed to detect associations of OS and RFS with anatomy-/staining-derived variables; hazard ratios (HR) and corresponding 95% confidence intervals (CI) were estimated. The prognostic effect in respect of OS and RFS was illustrated by Kaplan-Meier estimates; group differences were tested using the log-rank test. Correlation analysis was performed using Pearson’s correlation for log2 transformed data sets. A bubble plot was created using the Spotfire software on the basis of the correlation matrix. SPSS software version 24 (IBM Corporation, Armonk, New York, NY, USA) was used for statistical analyses; all *p*-values were given as two-sided, and *p* ≤ 0.05 was considered statistically significant.

## 5. Conclusions

Implementation of an integrative strategy named by us as DIICO enabled the detailed characterization of the anatomical features, cellular composition, proliferative and functional activity of lymphoid structures at three locations for patients with CRCLM. Knowledge derived from this exploratory study allowed to nominate the immune phenotype of ILSs in non-tumorous colon mucosa as the critical determinant of disease outcome. An in-depth understanding of the complexity of functional ILS/ELS may lead to new directions in therapeutic interventions and/or provide help in treatment decisions as part of personalized medicine and person-centered care. The power of the herein presented promising findings will be strengthened by the investigation of a larger, independent cohort of patients.

## Supplementary Materials

The following are available online at https://www.mdpi.com/2072-6694/12/11/3117/s1, Figure S1: Immune phenotype of lymphoid structures with GCs in tonsil tissue in comparison to ILSs of the colon, Figure S2: Inter-patient variability of lymphoid structure-associated immunological imprint of NT, CRC, and CRCLM, Figure S3: Expression of CD27 within lymphoid structures in tonsil tissues and NT, CRC, and CRCLM, Table S1: Description of anatomy - and staining-based variables, Table S2: Median and range of anatomy - and staining-based variables, Table S3: Correlation matrix for ILS/ELS-associated anatomy - and staining-derived variables, Table S4: Univariate Cox Regression analysis of ILS/ELS-associated anatomy - and staining-derived variables for RFS and OS, Table S5: Clinicopathological characteristics of patient cohort.
