# Supplementary material for: The Immune Phenotype of Isolated Lymphoid Structures in Non-Tumorous Colon Mucosa Encrypts the Information on Pathobiology of Metastatic Colorectal Cancer
Source: Cancers (Basel). 2020 Oct 25;12(11):3117. doi: 10.3390/cancers12113117 (PMC7692185; doi:10.3390/cancers12113117)
Supplement: Supplementary file 1 [file cancers-12-03117-s001.pdf]

Table S1. Description of anatomy- and staining-based variables.

Variable	Description	Type	n
**NT_ILS count**	number of ILS detected within NT specimen	anatomy-based	23
**NT_ILS size**	mean size (mm^2) of ILS detected within NT specimen	anatomy-based	23
**NT_ILS density**	mean cellular density (cells/mm^2) of ILS detected within NT specimen	anatomy-based	23
**NT_ILS to ILS distance**	mean distance (mm) between ILS detected within NT specimen	anatomy-based	22
**NT_AID$^+$ ILS count**	number of AID$^+$ ILS detected within NT specimen	staining-based	23
**NT_% GC size of ILS size**	mean proportion of GC area to the entire ILS area (%) in NT specimen	anatomy- and staining-based	14
**NT_% CD20$^+$ cells in ILS**	mean % of CD20$^+$ cells within ILS in NT specimen	staining-based	23
**NT_% Ki67$^+$ cells in ILS**	mean % of Ki67$^+$ cells within ILS in NT specimen	staining-based	23
**NT_% CD27$^+$ cells in ILS**	mean % of CD27$^+$ cells within ILS in NT specimen	staining-based	23
**CRC_ELS count**	number of ELS detected within CRC specimen	anatomy-based	21
**CRC_ELS size**	mean size (mm^2) of ELS detected within CRC specimen	anatomy-based	20
**CRC_ELS density**	mean cellular density (cells/mm^2) of ELS detected within CRC specimen	anatomy-based	20
**CRC_AID$^+$ ELS count**	number of AID$^+$ ELS detected within CRC specimen	staining-based	20
**CRC_% GC size of ELS size**	mean proportion of GC area to the entire ILS area (%) in CRC specimen	anatomy- and staining-based	9
**CRC_% CD20$^+$ cells in ELS**	mean % of CD20$^+$ cells within ELS in CRC specimen	staining-based	20
**CRC_% Ki67$^+$ cells in ELS**	mean % of Ki67$^+$ cells within ELS in CRC specimen	staining-based	20
**CRC_% CD27$^+$ cells in ELS**	mean % of CD27$^+$ cells within ELS in CRC specimen	staining-based	20
**CRCLM_ELS count**	number of ELS detected within CRCLM specimen	anatomy-based	22
**CRCLM_ELS size**	mean size (mm^2) of ELS detected within CRCLM specimen	anatomy-based	17
**CRCLM_ELS density**	mean cellular density (cells/mm^2) of ELS detected within CRCLM specimen	anatomy-based	17
**CRCLM_AID$^+$ ELS count**	number of AID$^+$ ILS detected within CRCLM specimen	staining-based	17
**CRCLM_% CD20$^+$ cells in ELS**	mean % of CD20$^+$ cells within ELS in CRCLM specimen	staining-based	17
**CRCLM_% Ki67$^+$ cells in ELS**	mean % of Ki67$^+$ cells within ELS in CRCLM specimen	staining-based	16
**CRCLM_% CD27$^+$ cells in ELS**	mean % of CD27$^+$ cells within ELS in CRCLM specimen	staining-based	16

Table S2. Median and range of anatomy- and staining-based variables

Variables \ Tissue	NT		CRC		CRCLM	
	median (range)	n	median (range)	n	median (range)	n
ILS/ELS count [number]	7 (1-25)	23	7 (0-18)	21	2 (0-21)	22
ILS/ELS size [mm^2]	0.139 (0.013-0.242)	23	0.105 (0.052-0.207)	20	0.115 (0.055-0.395)	17
ILS/ELS density [cells/mm^2]	13093.46 (11771.46-14140.52)	23	12443.58 (10894.95-15603.35)	20	12930.78 (10875.66-13799.11)	17
ILS to ILS distance [mm]	3.76 (1.35-8.68)	22	n.d.		n.d.	
AID^+ ILS/ELS count [number]	1 (0-9)	23	0 (0-6)	21	0 (0-2)	17
GC size of ILS/ELS size [%]	13.04 (1.55-29.28)	14	7.15 (0.57-17.05)	9	n.d.	
$CD20^+$ cells in ILS/ELS [%]	70.19 (29.30-94.04)	23	64.87 (29.33-83.64)	20	19.86 (5.69-75.94)	17
$Ki67^+$ cells in ILS/ELS [%]	5.40 (1.28-20.71)	23	2.26 (0.33-9.75)	20	1.76 (0.03-3.95)	16
$CD27^+$ cells in ILS/ELS [%]	77.34 (52.04-89.98)	23	73.05 (28.57-95.68)	20	68.33 (38.75-89.38)	16

n.d., not determined

Table S3. Correlation matrix for ILS/ELS-associated anatomy- and staining-derived variables

	NT_ILS count	NT_ILS size	NT_ILS density	NT_ILS to ILS distance	NT_AID⁺ ILS count	NT_% GC size to ILS size	NT_% CD20⁺ cells in ILS	NT_% Ki67⁺ cells in ILS	NT_% CD27⁺ cells in ILS	CRC_ELS count	CRC_ELS size	CRC_ELS density	CRC_AID⁺ ELS count	CRC_% GC size of ELS size	CRC_% CD20⁺ cells in ELS	CRC_% Ki67⁺ cells in ELS	CRC_% CD27⁺ cells in ELS	CRCLM_ELS count	CRCLM_ELS size	CRCLM_ELS density	CRCLM_AID⁺ ELS count	CRCLM_% CD20⁺ cells in ELS	CRCLM_% Ki67⁺ cells in ELS
NT_ILS size	0.214																						
	0.326																						
NT_ILS density	0.236	0.410																					
	0.278	0.052																					
NT_ILS to ILS distance	**-0.459**	0.361	-0.387																				
	**0.032**	0.099	0.075																				
NT_AID⁺ ILS count	0.341	0.348	**0.514**	-0.166																			
	0.111	0.104	**0.012**	0.461																			
NT_% GC size to ILS size	-0.477	0.108	0.170	0.046	-0.167																		
	0.084	0.713	0.561	0.875	0.569																		
NT_% CD20⁺ cells in ILS	-0.109	-0.358	-0.336	-0.169	-0.254	-0.004																	
	0.620	0.093	0.116	0.452	0.243	0.989																	
NT_% Ki67⁺ cells in ILS	-0.193	0.221	0.326	-0.114	0.405	0.459	0.075																
	0.378	0.311	0.129	0.613	0.055	0.099	0.733																
NT_% CD27⁺ cells in ILS	0.093	-0.030	-0.157	0.084	0.117	-0.451	0.319	-0.206															
	0.674	0.893	0.476	0.710	0.596	0.106	0.138	0.347															
CRC_ELS count	-0.061	-0.106	0.286	-0.131	0.252	0.081	-0.221	0.224	0.134														
	0.794	0.648	0.209	0.582	0.271	0.793	0.335	0.329	0.561														
CRC_ELS size	**0.506**	0.126	-0.164	-0.099	0.267	-0.431	-0.144	-0.118	-0.065	-0.212													
	**0.023**	0.598	0.490	0.688	0.256	0.162	0.545	0.621	0.786	0.370													
CRC_ELS density	0.094	-0.207	-0.369	0.133	-0.230	-0.313	0.351	-0.081	-0.067	**-0.472**	0.182												
	0.693	0.380	0.109	0.588	0.329	0.322	0.129	0.734	0.779	**0.036**	0.442												
CRC_AID⁺ ELS count	0.230	-0.196	0.333	-0.183	0.365	0.044	-0.249	0.305	-0.164	**0.548**	0.153	0.036											
	0.315	0.395	0.140	0.439	0.104	0.886	0.277	0.179	0.477	**0.010**	0.521	0.879											
CRC_% GC size of ELS size	-0.211	0.255	0.087	0.325	-0.120	-0.257	-0.499	-0.157	-0.040	-0.020	-0.469	-0.129	0.516										
	0.587	0.508	0.823	0.433	0.759	0.578	0.171	0.687	0.919	0.960	0.202	0.741	0.155										
CRC_% CD20⁺ cell in ELS	-0.146	-0.009	-0.047	0.082	-0.352	0.414	**0.507**	0.178	-0.119	-0.028	-0.144	0.228	0.154	0.252									
	0.538	0.970	0.845	0.737	0.128	0.181	**0.023**	0.454	0.618	0.908	0.543	0.334	0.517	0.514									
CRC_% Ki67⁺ cells in ELS	0.366	**-0.546**	-0.227	-0.287	-0.063	-0.006	-0.007	-0.227	-0.259	0.156	0.281	0.321	**0.483**	-0.392	-0.086								
	0.112	**0.013**	0.335	0.234	0.793	0.986	0.978	0.336	0.271	0.512	0.230	0.168	**0.031**	0.297	0.719								
CRC_% CD27⁺ cells in ELS	0.130	-0.229	-0.254	-0.009	0.345	-0.372	0.279	0.020	**0.701**	0.053	0.281	0.238	0.012	-0.395	-0.312	0.138							
	0.584	0.332	0.279	0.971	0.136	0.234	0.234	0.932	**< 0,001**	0.825	0.230	0.311	0.960	0.293	0.180	0.562							
CRCLM_ELS count	0.218	**0.452**	0.122	-0.047	0.304	0.287	0.231	**0.600**	-0.178	-0.191	0.146	0.178	0.110	-0.109	0.306	-0.194	-0.144						
	0.330	**0.035**	0.588	0.840	0.169	0.341	0.300	**0.003**	0.427	0.420	0.550	0.466	0.644	0.780	0.202	0.427	0.556						
CRCLM_ELS size	0.101	-0.061	-0.068	0.409	0.154	0.174	-0.331	-0.393	-0.011	-0.315	0.297	0.474	0.000	-0.213	-0.355	0.277	0.384	-0.348					
	0.701	0.815	0.794	0.103	0.554	0.609	0.194	0.119	0.967	0.253	0.303	0.087	0.999	0.646	0.213	0.337	0.175	0.171					
CRCLM_ELS density	-0.111	0.154	-0.412	-0.028	0.250	0.366	0.479	0.426	0.253	-0.305	0.364	0.101	-0.244	-0.506	0.120	-0.030	0.524	**0.571**	-0.040				
	0.671	0.555	0.101	0.915	0.333	0.268	0.052	0.088	0.327	0.269	0.201	0.732	0.380	0.247	0.683	0.919	0.054	**0.017**	0.879				
CRCLM_AID⁺ ELS count	0.318	-0.330	-0.084	-0.450	0.100	0.031	0.171	0.115	0.155	0.185	-0.144	-0.264	-0.001	-0.006	-0.084	0.181	0.111	**0.653**	-0.179	0.079			
	0.213	0.196	0.747	0.070	0.702	0.928	0.511	0.661	0.552	0.509	0.624	0.362	0.997	0.989	0.774	0.537	0.705	**0.004**	0.491	0.762			
CRCLM_% CD20⁺ cells in ELS	0.220	-0.025	-0.055	**-0.496**	0.131	-0.134	0.412	**0.506**	0.279	0.034	0.055	-0.352	0.045	-0.154	0.171	0.151	0.172	**0.588**	**-0.615**	0.391	0.444		
	0.397	0.925	0.833	**0.043**	0.615	0.693	0.101	**0.038**	0.278	0.903	0.851	0.217	0.873	0.742	0.559	0.606	0.557	**0.013**	**0.009**	0.121	0.074		
CRCLM_% Ki67⁺ cells in ELS	-0.048	0.112	0.101	0.059	0.036	**0.643**	-0.198	0.079	-0.453	-0.466	-0.364	-0.039	-0.481	-0.288	0.094	0.082	-0.419	0.182	0.265	0.189	0.192	-0.331	
	0.858	0.680	0.709	0.827	0.894	**0.045**	0.462	0.772	0.078	0.093	0.221	0.899	0.082	0.580	0.761	0.791	0.154	0.499	0.322	0.482	0.476	0.210	
CRCLM_% CD27⁺ cells in ELS	-0.042	-0.329	**-0.502**	-0.198	-0.165	-0.096	**0.627**	0.053	**0.673**	-0.105	0.049	0.052	-0.298	0.154	0.225	-0.455	0.467	0.296	-0.331	0.391	0.329	0.384	-0.165
	0.876	0.214	**0.047**	0.462	0.541	0.792	**0.009**	0.846	**0.004**	0.722	0.873	0.867	0.301	0.771	0.459	0.119	0.108	0.266	0.210	0.134	0.214	0.142	0.541

Correlation matrix including Pearson's correlation coefficient and the corresponding p values are shown.

Color code for statistically significant co-regularities; red for significant correlation coefficient≥ 0.5; blue for significant correlation coefficient < 0.5; statistically significant values are highlighted in bold.

Table S4. Univariate Cox Regression analysis of ILS/ELS-associated anatomy- and staining-derived variables for RFS and OS

	**RFS**		**OS**	
**Variable**	**p**	**HR (95% CI)**	**p**	**HR (95% CI)**
NT_ILS count	0.829	0.944 (0.559 - 1.593)	0.350	1.487 (0.647 - 3.417)
NT_ILS size	0.370	0.799 (0.490 - 1.304)	0.875	1.095 (0.354 - 3.382)
NT_ILS density	0.992	1.042 (2.6E-4 - 4.17E+03)	0.505	47.251 (5.7E-4 - 3.90E+6)
NT_ILS to ILS distance	0.522	1.356 (0.534 - 3.441)	0.903	0.929 (0.285 - 3.034)
NT_ILS AID^+ count	0.261	0.859 (0.658 - 1.120)	0.665	0.922 (0.640 - 1.329)
NT_% GC size of ILS size	0.270	0.648 (0.300 - 1.399)	0.890	0.925 (0.305 - 2.807)
NT_% $CD20^+$ cells in ILS	**0.043**	**0.309 (0.099 - 0.966)**	**0.044**	**0.222 (0.051 - 0.957)**
NT_% $Ki67^+$ cells in ILS	**0.016**	**0.450 (0.235 - 0.864)**	**0.023**	**0.314 (0.115 - 0.854)**
NT_% $CD27^+$ cells in ILS	0.934	0.885 (0.049 - 15.871)	0.713	0.501 (0.013 - 19.888)
CRC_ELS count	0.303	1.275 (0.803 - 2.025)	0.311	0.806 (0.531 - 1.224)
CRC_ELS size	0.664	0.748 (0.201 - 2.778)	0.490	2.177 (0.239 - 19.866)
CRC_ELS density	0.972	1.124 (0.002 - 766.747)	0.677	0.111 (4E-6 - 3.33E+03)
CRC_ELS AID^+ count	0.636	0.929 (0.685 - 1.260)	0.976	1.006 (0.667 - 1.518)
CRC_% GC size of ELS size	0.665	1.182 (0.554 - 2.521)	0.601	1.405 (0.393 - 5.027)
CRC_% $CD20^+$ cells in ELS	**0.041**	**0.149 (0.024 - 0.923)**	0.269	0.259 (0.023 - 2.848)
CRC_% $Ki67^+$ cells in ELS	0.633	1.150 (0.648 - 2.040)	0.474	1.425 (0.541 - 3.754)
CRC_% $CD27^+$ cells in ELS	0.904	0.925 (0.263 - 3.252)	0.821	0.817 (0.143 - 4.668)
CRCLM_ELS count	**6.2E-4**	**0.629 (0.482 - 0.820)**	**0.037**	**0.652 (0.437 - 0.974)**
CRCLM_ELS size	0.114	2.540 (0.800 - 8.067)	0.211	778.954 (0.023 - 2.65E+07)
CRCLM_ELS density	**0.026**	**9.7E-5 (2.83E-8 - 0.330)**	0.279	0.006 (5.37E-07 - 64.063)
CRCLM_ELS AID^+ count	0.546	0.435 (0.029 - 6.456)	0.681	0.452 (0.010 - 20.107)
CRCLM_% $CD20^+$ cells in ELS	**0.021**	**0.166 (0.036 - 0.764)**	0.132	0.238 (0.037 - 1.541)
CRCLM_% $Ki67^+$ cells in ELS	0.587	1.192 (0.633 - 2.244)	0.452	1.987 (0.332 - 11.906)
CRCLM_% $CD27^+$ cells in ELS	0.171	0.210 (0.023 - 1.956)	0.093	0.092 (0.006 - 1.482)

Statistically significant results ($p < 0.05$) are highlighted in bold. The median and range of the variables is given in Table S2.

Table S5. Clinicopathological characteristics of patient cohort.

**Patients (%)**	**23 (100)**
Age at surgery [years] (2 missing [8.7 %])	
Median (range)	65.35 (45.76-78.55)
Sex	
Male	14 (60.9)
Female	9 (39.1)
T stage of primary tumor (2 missing [8.7 %])	
T2	6 (26.1)
T3	14 (60.9)
T4	1 (4.3)
N stage of primary tumor (2 missing [8.7 %])	
node negative	6 (26.1)
node positive	15 (65.2)
M stage of primary tumor (1 missing [4.3 %])	
M0	16 (69.6)
M1	6 (26.1)
Median number of metastases (1 missing [4.3 %])	
Median (range)	2 (1-10)
Largest median diameter [cm] (1 missing [4.3 %])	
Median (range)	2.4 (0.5-9.0)
Disease free interval [months] (4 missing [17.4 %])	
Median (range)	16.8 (0-117.20)

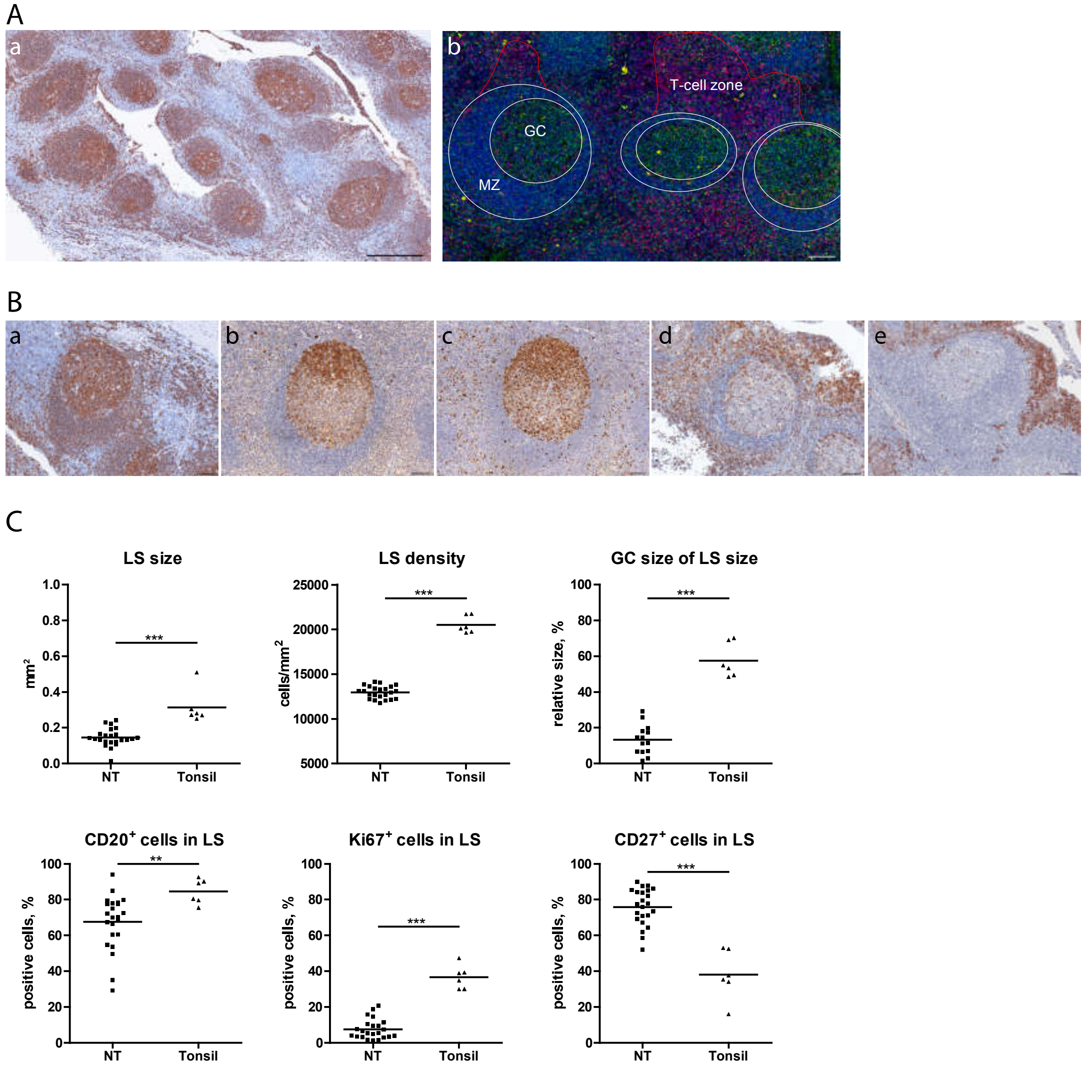

Figure S1. Immune phenotype of lymphoid structures with GCs in tonsil tissue in comparison to ILS of the colon. (A) Shown is overall view of the tonsil tissue with multiple GCs upon IHC staining for CD20 (a, color code: brown, CD20; blue, nuclear counterstaining with hematoxylin; scale bar: 500 µm) and IF double staining for the CD20/CD3 combination (b, color code: green, CD20; red, CD3; blue, nuclear counterstaining by DAPI; scale bar: 100 µm). Germinal center (GC) and mantle zone (MZ) as well as the T-cell zone is indicated. (B) Representative images of tonsil tissue sections stained for the markers CD20 (a), AID (b), Ki67 (c), CD27 (d), and CD138 (e). Color code: brown, the marker; blue, nuclear counterstaining with hematoxylin. Scale bar: 100 µm. (C) Vertical scatter plots with mean illustrate the anatomy- and staining-derived variables for lymphoid structures within NT (number of specimens included into the individual analyses see Table S2) and tonsil (n = 6, with 5 lymphoid structures per tonsil tissue). Significant differences are indicated by asterisks ** ($p < 0.01$) and *** ($p < 0.001$) (Mann-Whitney-U test). Herein, both ILS in NT and lymphoid structures in tonsil tissue are referred to as LS.

Figure S1

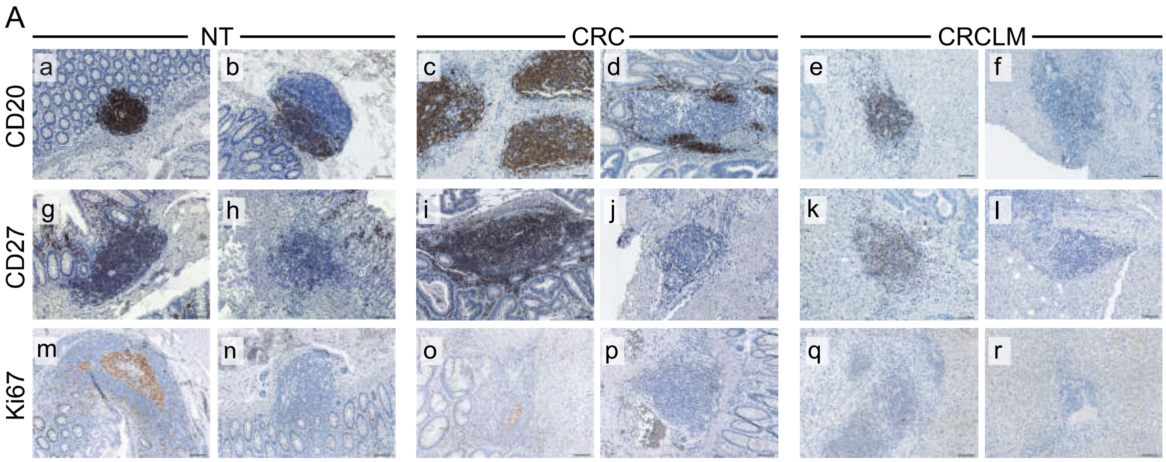

Figure S2. Inter-patient variability of lymphoid structure-associated immunological imprint of NT, CRC, and CRCLM. Representative images of ILS and ELS within tissue sections stained for CD20, CD27 or Ki67 (brown color) and counterstained for nuclei with hematoxylin (blue color) are shown for three tissue entities (NT, CRC and CRCLM). Shown are specimens with lymphoid structures characterized by high (a, c, e, g, i, k, m o, q) or low (b, d, f, h, j, l, n, p, r) magnitude of the corresponding cell population. Scale bar: 100 µm. Corresponding variables are described with median, minimum and maximum in Table S2.

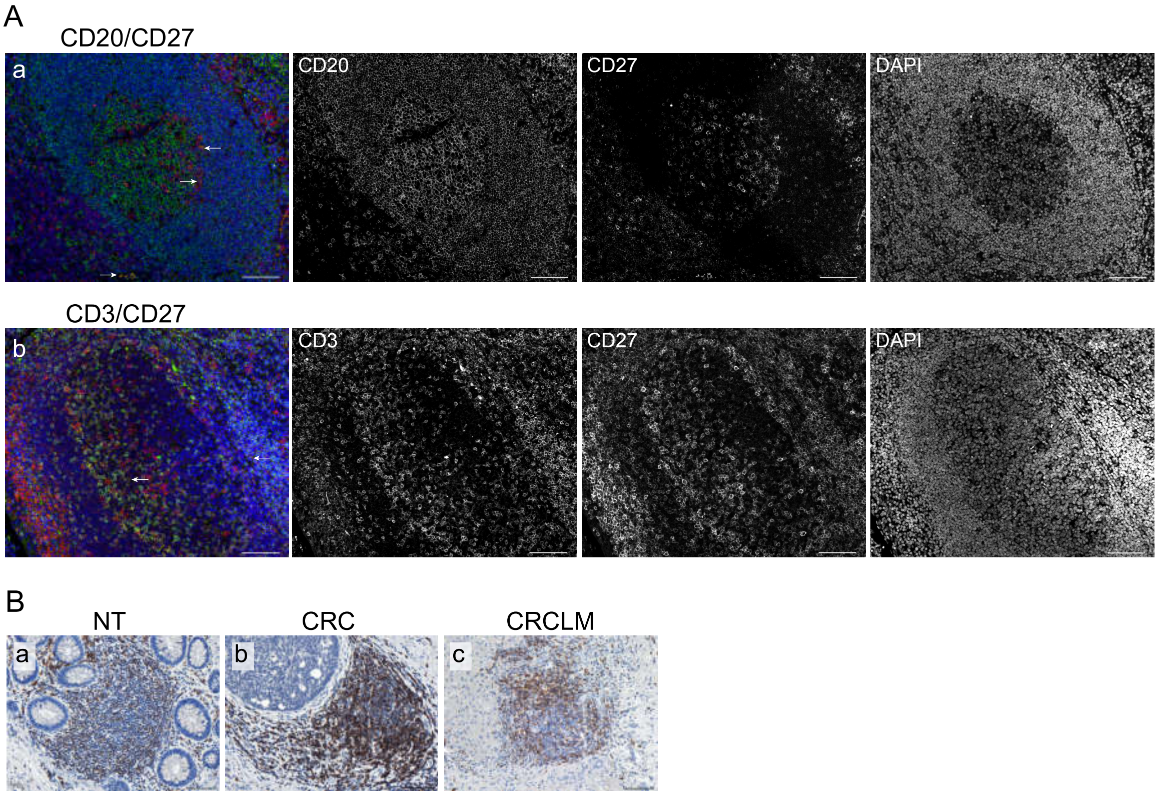

Figure S3. Expression of CD27 within lymphoid structures in tonsil tissues and NT, CRC, and CRCLM. (A) Localization of CD20/CD27 double-positive B cells (a) and CD3/CD27 double-positive T cells (b) within lymphoid structures of tonsil tissues assessed by IF staining. Color code: green, CD20 or CD3; red, CD27; blue, nuclear counterstaining by DAPI. Shown are images for individual channels in a black/white mode and the merged images in color. Examples of double-positive cells are indicated by white arrows. Scale bar: 100 µm. (B) Representative images of ILS and ELS within tissue sections stained for CD27 (brown color) and counterstained for nuclei with hematoxylin (blue color) are shown at three tissue entities (a, NT; b, CRC; c, CRCLM). Scale bar: 100 µm.

Figure S3
